# Generalizations of the ‘Linear Chain Trick’: incorporating more flexible dwell time distributions into mean field ODE models

**DOI:** 10.1007/s00285-019-01412-w

**Published:** 2019-08-13

**Authors:** Paul J. Hurtado, Adam S. Kirosingh

**Affiliations:** 1 University of Nevada, Reno, Reno, USA; 2 Stanford University, Stanford, USA

**Keywords:** Gamma chain trick, Linear Chain Trick, Distributed delay, Mean field model, Phase-type distributions, Time lag, 37N25, 92B05, 92D25, 92D30, 92D40, 45D05, 60J28

## Abstract

In this paper we generalize the Linear Chain Trick (LCT; aka the Gamma Chain Trick) to help provide modelers more flexibility to incorporate appropriate dwell time assumptions into mean field ODEs, and help clarify connections between individual-level stochastic model assumptions and the structure of corresponding mean field ODEs. The LCT is a technique used to construct mean field ODE models from continuous-time stochastic state transition models where the time an individual spends in a given state (i.e., the dwell time) is Erlang distributed (i.e., gamma distributed with integer shape parameter). Despite the LCT’s widespread use, we lack general theory to facilitate the easy application of this technique, especially for complex models. Modelers must therefore choose between constructing ODE models using heuristics with oversimplified dwell time assumptions, using time consuming derivations from first principles, or to instead use non-ODE models (like integro-differential or delay differential equations) which can be cumbersome to derive and analyze. Here, we provide analytical results that enable modelers to more efficiently construct ODE models using the LCT or related extensions. Specifically, we provide (1) novel LCT extensions for various scenarios found in applications, including conditional dwell time distributions; (2) formulations of these LCT extensions that bypass the need to derive ODEs from integral equations; and (3) a novel Generalized Linear Chain Trick (GLCT) framework that extends the LCT to a much broader set of possible dwell time distribution assumptions, including the flexible *phase-type* distributions which can approximate distributions on ${\mathbb {R}}^+$ and can be fit to data.

## Introduction

Many scientific applications involve systems that can be framed as continuous time state transition models (e.g., see Strogatz 2014), and these are often modeled using mean field ordinary differential equations (ODE) of the form

1 $$\begin{aligned} \frac{d{\mathbf {x}}}{dt}=f({\mathbf {x}},\varvec{\theta },t), \end{aligned}$$

where ${\mathbf {x}}(t) \in {\mathbb {R}}^n$, parameters $\varvec{\theta }\in {\mathbb {R}}^p$, and $f:\; {\mathbb {R}}^n \mapsto {\mathbb {R}}^n$ is smooth. The abundance of such applications, and the accessibility of ODE models in terms of the analytical techniques and computational tools for building, simulating, and analyzing ODE models [e.g., straightforward simulation methods and bifurcation analysis software like MatCont (Dhooge et al. 2003) and XPPAUT (Ermentrout 1987, 2019)], have made ODEs one of the most popular modeling frameworks in scientific applications.

Despite their widespread use, one shortcoming of ODE models is their inflexibility when it comes to specifying probability distributions that describe the duration of time individuals spend in a given state. The basic options available for assuming specific dwell time distributions within an ODE framework can really be considered as a single option: the 1st event time distribution for a (nonhomogeneous) Poisson process, which includes the exponential distribution as a special case.

To illustrate this, consider the following SIR model of infectious disease transmission by Kermack and McKendrick (1927),

2a $$\begin{aligned} \frac{d}{dt}S(t)&=\; -\lambda (t)\,S(t) \end{aligned}$$

2b $$\begin{aligned} \frac{d}{dt}I(t)&=\; \lambda (t)\,S(t) - \gamma \,I(t) \end{aligned}$$

2c $$\begin{aligned} \frac{d}{dt}R(t)&=\; \gamma \,I(t) \end{aligned}$$

where *S*(*t*), *I*(*t*), and *R*(*t*) correspond to the number of susceptible, infected, and recovered individuals in a closed population at time *t* and $\lambda (t)\equiv \beta \,I(t)$ is the per-capita infection rate (also called the *force of infection* by Anderson and May (1992) and others). This model can be thought of as the mean field model for some underlying stochastic state transition model where a large but finite number of individuals transition from state S to I to R [see the Appendix, Kermack and McKendrick (1927) for a derivation, and see Armbruster and Beck (2017), Banks et al. (2013), and references therein for examples of the convergence of stochastic models to mean field ODEs].

Although multiple stochastic models can yield the same mean field deterministic model, it is common to consider a stochastic model based on Poisson processes. For the SIR model above, for example, this stochastic analog would assume that, over the time interval $[t,t+{\varDelta } t]$ (for very small ${\varDelta } t$), an individual in state S or I at time *t* is assumed to transition from S to I with probability $\lambda (t)\,{\varDelta } t$, or from I to R with probability $\gamma \,{\varDelta } t$, respectively. Taking ${\varDelta } t \rightarrow 0$ yields the desired continuous time stochastic model. Here, the linear rate of transitions from I to R ($\gamma \,I(t)$) arises from assuming the dwell time for an individual in the infected state (I) follows an exponential distribution with rate $\gamma$ (i.e., the 1st event time distribution for a homogeneous Poisson process with rate $\gamma$). Similarly, assuming the time spent in state S follows the 1st event time distribution under a nonhomogeneous (also called inhomogeneous) Poisson process with rate $\lambda (t)$ yields a time-varying per capita transition rate $\lambda (t)$. This association of a mean field ODE with a specific underlying stochastic model provides very valuable intuition in an applied context. For example, it allows modelers to ascribe application-specific (e.g., biological) interpretations to parameters and thus estimate parameter values (e.g., for $\gamma$ above, the mean time spent infectious is $1/\gamma$), and it provides intuition and a clear mathematical foundation from which to construct and evaluate mean field ODE models based on individual-level, stochastic assumptions.

To construct similar models using other dwell time distributions, a standard approach is to formulate a continuous time stochastic model and from it derive mean field *distributed delay equations*, typically represented as integro-differential equations (IDEs) or integral equations (IEs) (e.g., see Kermack and McKendrick 1927; Hethcote and Tudor 1980; Feng et al. 2007, 2016, or see the example derivation in the Appendix). Readers unfamiliar with IEs and IDEs are referred to Burton (2005) or similar texts. IEs and IDEs have proven to be quite useful models in biology, e.g., they have been used to model chemical kinetics (Roussel 1996), gene expression (Smolen et al. 2000; Takashima et al. 2011; Guan and Ling 2018), physiological processes such as glucose-insulin regulation (Makroglou et al. 2006, and references therein), cell proliferation and differentiation (Özbay et al. 2008; Clapp and Levy 2015; Yates et al. 2017), cancer biology and treatment (Piotrowska and Bodnar 2018; Krzyzanski et al. 2018; Câmara De Souza et al. 2018), pathogen and immune response dynamics (Fenton et al. 2006), infectious disease transmission (Anderson and Watson 1980; Lloyd 2001a, b; Feng and Thieme 2000; Wearing et al. 2005; Lloyd 2009; Feng et al. 2007; Ciaravino et al. 2018), and population dynamics (MacDonald 1978a; Blythe et al. 1984; Metz and Diekmann 1986; Boese 1989; Nisbet et al. 1989; Cushing 1994; Wolkowicz et al. 1997; Gyllenberg 2007; Wang and Han 2016; Lin et al. 2018; Robertson et al. 2018). See also Campbell and Jessop (2009) and the applications reviewed therein.

However, while distributed delay equations are very flexible, in that they can incorporate arbitrary dwell time distributions, when compared to ODEs they also can be more challenging to derive, to analyze mathematically, and to simulate (Cushing 1994; Burton 2005). Thus, many modelers face a trade-off between building appropriate dwell time assumptions into their mean field models (i.e., opting for an IE or IDE model) and constructing parsimonious models that are more easily analyzed both mathematically and computationally (i.e., opting for an ODE model). For example, the following system of integral equations generalizes the SIR example above by incorporating an arbitrary distribution for the duration of infectiousness (i.e., the dwell time in state I):

3a $$\begin{aligned} S(t)&=\; S(0)(1-F_S(t)) \end{aligned}$$

3b $$\begin{aligned} I(t)&=\; I(0)(1-F_I(t)) + \int _0^t \beta \,I(u)\,S(u)\,(1-F_I(u))\,du \end{aligned}$$

3c $$\begin{aligned} R(t)&=\; N - S(t) - I(t) \end{aligned}$$

where $N=S(0)+I(0)+R(0)$, $1-F_S(t)=\exp \big (-\int _0^t\beta \,I(u)\,du\big )$ is the survival function for the distribution of time spent in susceptible state S [i.e., the 1st event time under a Poisson process with rate $\lambda (t)=\beta \,I(t)$], and $1-F_I(t)=\exp \big (-\gamma \,t\big )$ is the survival function for the time spent in the infected state I (related models can be found in, e.g., Feng and Thieme 2000; Ma and Earn 2006; Krylova and Earn 2013; Champredon et al. 2018). A different choice of the CDF $F_I$ allows us to generalize the SIR model to other dwell time distributions that describe the time individuals spend in the infected state. Integral equations like those above can also be differentiated (assuming the integrands are differentiable) and represented as integrodifferential equations (e.g., as in Hethcote and Tudor 1980).

There have been some efforts in the past to identify which categories of integral and integro-differential equations can be reduced to systems of ODEs (e.g., MacDonald 1989; Metz and Diekmann 1991; Ponosov et al. 2002; Jacquez and Simon 2002; Burton 2005; Goltser and Domoshnitsky 2013; Diekmann et al. 2017, and references therein), but in practice the most well known case is the reduction of IEs and IDEs that assume Erlang^1^ distributed dwell times. This is done using what has become known as the Linear Chain Trick (LCT, also referred to as the Gamma Chain Trick; MacDonald 1978b; Smith 2010) which dates at least back to Fargue (1973) and earlier work by Theodore Vogel (e.g., Vogel 1961, 1965, according to Câmara De Souza et al. 2018). However, for more complex models that exceed the level of complexity that can be handled by existing “rules of thumb” like the LCT, the current approach is to derive mean field ODEs from mean field integral equations that might themselves first need to be derived from system-specific stochastic state transition models (e.g., Kermack and McKendrick 1927; Feng et al. 2007; Banks et al. 2013; Feng et al. 2016, and see the Appendix for an example.). Unfortunately, modelers often avoid these extra (often laborious) steps in practice by assuming (sometimes only implicitly) very simplistic dwell time distributions based on Poisson process 1st event times as in the SIR example above.

In light of the widespread use of ODE models, these challenges and trade-offs underscore a need for a more rigorous theoretical foundation to more effectively and more efficiently construct mean field ODE models that include more flexible dwell time distribution assumptions (Wearing et al. 2005; Feng et al. 2016; Robertson et al. 2018). The goal of this paper is to address these needs by (1) providing a theoretical foundation for constructing the desired system of ODEs directly from “first principles” (i.e., stochastic model assumptions), without the need to derive ODEs from intermediate IDEs or explicit stochastic models, and by (2) providing similar analytical results for novel extensions of the LCT which allow more flexible dwell time distributions, and conditional relationships among dwell time distributions, to be incorporated into ODE models. We also aim to clarify how underlying (often implicit) stochastic model assumptions are reflected in the structure of corresponding mean field ODE model equations.

The remainder of this paper is organized as follows. An intuitive description of the Linear Chain Trick (LCT) is given in Sect. 1.1 as a foundation for the extensions that follow. In Sect. 2 we review key notation and properties of Poisson processes and certain probability distributions needed for the results that follow. In Sect. 3.1.1 we highlight the association between Poisson process intensity functions and per capita rates in mean field ODEs, and in Sect. 3.1.2 we introduce what we call the *weak memorylessness* property of (nonhomogeneous) Poisson process 1st event time distributions. In Sects. 3.2 and 3.3 we give a formal statement of the LCT and in Sect. 3.4 a generalization that allows time-varying rates in the underlying Poisson processes. We then provide similar LCT generalizations for more complex cases: In Sect. 3.5 we provide results for multiple ways to implement transitions from one state to multiple states (which arise from different stochastic model assumptions and lead to different systems of mean field ODEs), and we address dwell times that obey Erlang mixture distributions. In Sect. 3.6 we detail how to construct mean field ODEs in which a sub-state transition (e.g., from infected to quarantined) does or does not alter the overall dwell time distribution in that state (e.g., the duration of infection). Lastly, in Sect. 3.7 we present a Generalized Linear Chain Trick (GLCT) which describes how to construct mean field ODEs from first principles based on assuming a very flexible family of dwell time distributions that include the *phase-type* distributions, i.e., hitting time distributions for continuous time Markov chains (Reinecke et al. 2012a; Horváth et al. 2016) which we address more specifically in Sect. 3.7.1. Tools for fitting phase-type distributions to data, or using them to approximate other distributions, are also mentioned in Sect. 3.7.1.

### Intuitive description of the Linear Chain Trick

To begin, an intuitive understanding of the Linear Chain Trick (LCT) based on properties of Poisson processes, is helpful for drawing connections between underlying stochastic model assumptions and the structure of corresponding mean field ODEs. Here we consider a very basic case (detailed in Sect. 3.2): the mean field ODE model for a stochastic process in which particles in state X remain there for an Erlang(*r*, *k*) distributed amount of time before exiting to some other state.

In short, the LCT exploits a natural stage structure within state X imposed by assuming an Erlang(*r*, *k*) distributed dwell time [i.e., a gamma(*r*, *k*) distribution with integer shape *k*]. Recall that the time to the *k*th event under a homogeneous Poisson process with rate $r>0$ is Erlang(*r*, *k*) distributed. In that context, each event is preceded by a length of time that is exponentially distributed with rate *r*, and thus the time to the *k*th event is the sum of *k* independent and identically distributed exponential random variables [i.e., the sum of *kiid* exponential random variables with rate *r* is Erlang(*r*, *k*) distributed]. Particles in state X at a given time can therefore be classified by which event they are awaiting next, i.e., a particle is in state X$_i$ if it is waiting for the *i*th event to occur, and X$_1$, $\ldots$, X$_k$ is a partition of X. The dwell time distribution for each sub-state X$_i$ is exponential with rate *r*, and particles leave the last state X$_k$ (and thus X) upon the occurrence of the *k*th event.

This sub-state partition is useful to impose on X because the corresponding mean field equations for these sub-states are linear (or nearly linear) ODEs. Specifically, if $x_i(t)$ is the amount in X$_i$ at time *t*, these mean field ODEs are

4 $$\begin{aligned} \begin{aligned} \frac{d}{dt}x_1(t)&= -r\,x_1(t), \\ \frac{d}{dt}x_i(t)&= r\,x_{i-1}(t)\,-\,r\,x_i(t) \quad \text { for } i=2,\ldots ,k \end{aligned} \end{aligned}$$

where the total amount in X at time *t* is $x(t)=\sum _{i=1}^k x_i(t)$, $x_1(0)=x_0$ and $x_i(0)=0$ for $i=2,\ldots ,k$.

As we show below, this Poisson process based perspective allows us to generalize the LCT to other more complex scenarios where we partition a focal state X in a similar fashion. Those results are further extended from exponential dwell times to 1st event time distributions under a nonhomogeneous Poisson processes with time varying rate *r*(*t*), allowing for time-varying (or state-dependent) dwell time distributions to be used in extensions of LCT.

## Model framework

The context in which we consider applications of the Linear Chain Trick (LCT) is the derivation of continuous time mean field model equations for stochastic state transition models with a distributed dwell time in a focal state, X. Such mean field models might otherwise be modeled as integral equations (IEs) or integro-differential equations (IDEs), and we seek to identify generalizations of the LCT that allow us to replace mean field integral equations with equivalent systems of 1st order ODEs.

### Distributions and notation

Below we will extend the LCT from Erlang(*r*, *k*) distributions (i.e., *k*th event time distributions under homogeneous Poisson processes with rate *r*) to event time distributions under nonhomogeneous Poisson processes with time varying rate *r*(*t*), and related distributions like the minimum of multiple Erlang random variables. In this section we will first review some basic properties of these distributions.

Gamma distributions can be parameterized^2^ by two strictly positive quantities: *rater* and *shapek* (sometimes denoted $\alpha$ and $\beta$, respectively). The Erlang family of distributions are the subset of gamma distributions with integer-valued shape parameters $k\in \mathbb {Z_+}$, or equivalently, the distributions resulting from the sum of *kiid* exponential distributions. That is, if a random variable $T=\sum _{i=1}^k T_i$, where all $T_i$ are independent exponential distributions with rate *r*, then *T* is Erlang(*r*, *k*) distributed. Since the inter-event times under a homogeneous Poisson process are exponentially distributed, the time to the *k*th event is thus Erlang(*r*, *k*). This construction is foundational to a proper intuitive understanding of the LCT and its extensions.

If random variable *T* is gamma(*r*, *k*) distributed, then its mean $\mu$, variance $\sigma ^2$, and coefficient of variation $c_v$ are given by

5 $$\begin{aligned} \mu =\frac{k}{r} \text {, }\; \sigma ^2=\frac{k}{r^2} \;\text {, and }\; c_v=\frac{1}{\sqrt{k}}. \end{aligned}$$

Note that gamma distributions can be parameterized by their mean $\mu$ and variance $\sigma ^2$ by rewriting rate *r* and shape *k* using Eq. (5):

6 $$\begin{aligned} r = \frac{\mu }{\sigma ^2} \; \text {, and }\; k = \frac{\mu ^2}{\sigma ^2}. \end{aligned}$$

However, to ensure this gamma distribution is also Erlang (i.e., to ensure the shape parameter *k* is an integer) one must adjust the assumed variance up or down by rounding the value of *k* in Eq. (6) down or up, respectively, to the nearest integer (see Appendix B for details, and alternatives).

The Erlang density function (*g*), CDF (*G*), and survival^3^ function ($S=1-G$; also called the *complementary CDF*) are given by

7a $$\begin{aligned} g^k_r(t)&=\; r\,\frac{(r\,t)^{k-1}}{(k-1)!}e^{-rt} \end{aligned}$$

7b $$\begin{aligned} G^k_r(t)&=\; 1 - \sum _{j=1}^{k} \frac{(r\,t)^{j-1}}{(j-1)!}e^{-r\,t} = 1- \sum _{j=1}^{k} \frac{1}{r}\,g_r^j(t) \end{aligned}$$

7c $$\begin{aligned} S^k_r(t)&=\; 1-G^k_r(t) = \sum _{j=1}^{k} \frac{1}{r}\,g_r^j(t). \end{aligned}$$

The results in Sect. 3 use (and generalize) the property of Erlang distributions detailed in Lemma 1 (eqs. 7.11 in Smith 2010, restated here without proof), which is the linchpin of the LCT.

#### Lemma 1

Erlang density functions $g_r^j(t)$, with rate *r* and shape *j*, satisfy

8a $$\begin{aligned} \frac{d}{dt}g^1_{r}(t)&=\; -r\,g^1_{r}(t), \qquad \quad \qquad \quad \;\;\; \text { where }\; g^1_{r}(0)=r, \end{aligned}$$

8b $$\begin{aligned} \frac{d}{dt}g^j_{r}(t)&=\; r\,[\,g^{j-1}_{r}(t)-g^{j}_{r}(t)\,],\quad \text { where }\; g^j_{r}(0)=0 \text { for }j\ge 2. \end{aligned}$$

Since homogeneous Poisson processes are a special case of nonhomogeneous Poisson processes^4^ from here on we will use “Poisson process” or “Poisson process with rate *r*(*t*)” to refer to cases that apply to both homogeneous [i.e., $r(t)=r$ constant] and nonhomogeneous Poisson processes.

The more general *k*th event time distribution under a Poisson process with rate *r*(*t*), starting from some time $\tau < t$ has a density function ($h_{r}^k$), survival function (${\mathcal {S}}_{r}^k$), and CDF ($H_{r}^k\equiv 1-{\mathcal {S}}_{r}^k$) given by

9a $$\begin{aligned} h^k_{r}(t,\tau )&=\; r(t)\,\frac{m(t,\tau )^{k-1}}{(k-1)!}\,e^{-m(t,\tau )} \quad \text { and} \end{aligned}$$

9b $$\begin{aligned} {\mathcal {S}}^k_{r}(t,\tau )&=\;\sum _{j=1}^{k} \frac{h^j_{r}(t,\tau )}{r(t)} \quad \text {where} \end{aligned}$$

9c $$\begin{aligned} m(t,\tau )&\equiv \; \int _\tau ^t r(s)\,ds \quad \text {and} \quad \frac{d}{dt}m(t,\tau )=r(t). \end{aligned}$$

For an arbitrary survival function starting at time $\tau$ (i.e., over the period $[\tau ,t]$ where $t\ge \tau$) we use the notation $S(t,\tau )$. In some instances, we also use the notation $S(t)\equiv S(t,0)$.

Lastly, in the context of state transitions models, it is common to assume that, upon leaving a given state (e.g., state X) at time *t*, individuals are distributed across multiple recipient states according to a generalized Bernoulli distribution (also known as the *categorical distribution* or the multinomial distribution with 1 trials) defined on the integers 1 through *k* where the probability of a particle entering the *j*th of *k* recipient states ($j\in {1,\ldots ,k}$) is $p_j(t)$, which define probability vector ${\mathbf {p}}(t) = [p_1(t),\ldots ,p_k(t)]^\text {T}$ and $\sum _{j=1}^k p_j(t)=1$.

## Results

The results below focus on one or more states, within a potentially larger state transition model, for which we would like to assume a particular dwell time distribution and derive a corresponding system of mean field ODEs using the LCT or a generalization of the LCT. In particular, the results below describe how to construct those mean field ODEs directly from stochastic model assumptions without needing to derive them from equivalent mean field integral equations (which themselves might need to be derived from an explicit continuous-time stochastic model).

### Preliminaries

Before presenting extensions of the LCT, we first illustrate in Sect. 3.1.1 how mean field ODEs include terms that reflect underlying Poisson process rates. In Sect. 3.1.2, we highlight a key property of these Poisson process 1st event time distributions that we refer to as a *weak memorylessness property* since it is a generalization of the well known memorylessness property of the exponential and geometric distributions.

#### Transition rates in ODEs reflect underlying Poisson process rates

To build upon the intuition spelled out in Sect. 1.1, where particles exit X following an exponentially distributed dwell time, we now assume that particles exit X following the 1st event time under nonhomogeneous Poisson processes with rate *r*(*t*) (recall the 1st event time distribution is exponential if $r(t)=r$ is constant). As illustrated by the corresponding mean field equations given below, the rate function *r*(*t*) can be viewed as either the intensity function^5^ for the Poisson process governing when individuals leave state X, or as the (mean field) per-capita rate of loss from state X as shown in Eq. (11). This dual perspective provides valuable intuition for critically evaluating mean field ODEs.

##### Example 1

*(Equivalence between Poisson process rates and per capita rates in mean field ODEs)* Consider the scenario described above. The survival function for the dwell time distribution for a particle entering X at time $\tau$ is $S(t,\tau )=\exp (-\int _{\tau }^{t} r(u)\,du)$, and it follows that the expected proportion of such particles remaining in X at time $t>\tau$ is given by $S(t,\tau )$. Let *x*(*t*) be the total amount in state X at time *t*, $x(0)=x_0$, and assume that ${\mathcal {I}}(t)$ and *r*(*t*) are integrable, non-negative functions of *t*. Then the corresponding mean field integral equation for this scenario is

10 $$\begin{aligned} x(t) =\; x_0\,S(t,0) + \int ^t_{0} {\mathcal {I}}(\tau )\, S(t,\tau ) d\tau \end{aligned}$$

and the integral equation (10) above is equivalent to

11 $$\begin{aligned} \frac{d}{dt}{x}(t) =\; {\mathcal {I}}(t) - r(t)\, x(t), \; \text { with } x(0)=x_0. \end{aligned}$$

##### Proof

The Leibniz rule for differentiating integrals, Eq. (10), and Lemma 1 yield

12 $$\begin{aligned} \begin{aligned} \frac{d}{dt}{x}(t)&=\; x_0\frac{d}{dt}S(t,0) + \frac{d}{dt}\int ^t_{0} {\mathcal {I}}(\tau )\,S(t,\tau )d\tau \\&=\; -r(t)\,x_0\,e^{-\int _{0}^{t}r(u)\,du} +{\mathcal {I}}(t) -r(t) \int ^t_{0}{\mathcal {I}}(\tau ) e^{-\int _{\tau }^{t}r(u)\,du} d\tau \\&=\; {\mathcal {I}}(t) - r(t)\bigg [x_0\,e^{-\int _{0}^{t}r(u)\,du} + \int ^t_{0} {\mathcal {I}}(\tau )e^{-\int _{\tau }^{t}r(u)\,du} d\tau \bigg ] \\&=\; {\mathcal {I}}(t) - r(t) x(t). \end{aligned} \end{aligned}$$

$$\square $$

#### Weak memoryless property of Poisson process 1st event time distributions

The intuition behind the LCT, and the history-independent nature of ODEs, are related to the memorylessness property of exponential distributions. For example, when particles accumulate in a state with an exponentially distributed dwell time distribution, then at any given time all particles currently in that state have *iid* exponentially distributed amounts of time left before they leave that state regardless of the duration of time already spent in that state. Each of these can be viewed as a reflection of the history-independent nature of Poisson processes.

Accordingly, the familiar memorylessness property of exponential and geometric distributions can, in a sense, be generalized to (nonhomogeneous) Poisson process 1st event time distributions. Recall that if an exponentially distributed (rate *r*) random variable *T* represents the time until some event, then if the event has not occurred by time *s* the remaining duration of time until the event occurs is also exponential with rate *r*. The analogous *weak memorylessness* property of nonhomogeneous Poisson process 1st event time distributions is detailed in the following definition.

##### Definition 1

**(Weak memorylessness property of Poisson process 1st event times)** Assume *T* is a Poisson process 1st event time starting at time $\tau$, which has rate *r*(*t*) and CDF $H_r^1(t,\tau )=1-\exp (-m(t,\tau ))$ [see Eqs. (9) and (9c)]. If the event has not occurred by time $s>\tau$ the distribution of the remaining time $T_s \equiv T-s\;|\;T>s$ follows a shifted but otherwise identical Poisson process 1st event time distribution with CDF $P(T_s \le t)=H_r^1(t+s,s)$. If $r(t)=r$ is a positive constant we recover the memorylessness property of the exponential distribution.

##### Proof

The CDF of $T_s$ (for $t>\tau$) is given by

13 $$\begin{aligned} \begin{aligned} P(T_s \le t)&=\; P(T - s \le t \;|\; T>s) =\; \frac{P(s< T \le s+ t)}{P(s<T)} \\&=\; \frac{H_r^1(t+s,\tau ) - H_r^1(s,\tau )}{1-H_r^1(s,\tau )} =\; 1-\frac{1-H_r^1(t+s,\tau )}{1-H_r^1(s,\tau )}\\&=\; 1-\frac{e^{-m(t+s,\tau )}}{e^{-m(s,\tau )}} =\; 1-e^{-m(t+s,s)} =\; H_r^1(t+s,s). \\ \end{aligned} \end{aligned}$$

$$\square $$

In other words, Poisson process 1st event time distributions are memoryless up to a time shift in their rate functions. In the context of multiple particles entering a given state X at different times and leaving according to independent Poisson process 1st event times with identical rates *r*(*t*) (i.e., *t* is absolute time, not time since entry into X), then for all particles in state X at a given time the distribution of time remaining in state X is (1) independent of how much time each particle has already spent in X and (2) follows *iid* Poisson process 1st event time distributions with rate *r*(*t*).

### Simple case of the LCT

**Fig. 1:**
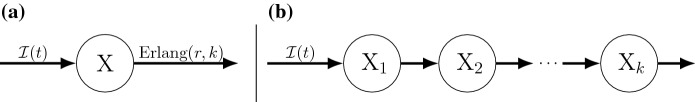
Example diagram where state X has an Erlang(*r*, *k*) distributed dwell time, represented either as **a** a single state and corresponding integral equation, or **b** as a set of *k* sub-states each with exponential dwell time distributions whose mean field equations can be represented as either integral equations or a system of ODEs (see Theorem 1). Rate ${\mathcal {I}}(t)$ is an integrable non-negative function describing the mean field influx rate into state X

To illustrate how the LCT follows from Lemma 1, consider the simple case of the LCT illustrated in Fig. 1, where a higher dimensional model includes a state transition into, then out of, a focal state X. Assume the time spent in that state ($T_X$) is Erlang(*r*, *k*) distributed [i.e., $T_X \sim$ Erlang(*r*, *k*)]. Then the LCT provides a system of ODEs equivalent to the mean field integral equations for this process as detailed in the following theorem:

#### Theorem 1

(Simple LCT) Consider a continuous time state transition model with inflow rate ${\mathcal {I}}(t)\ge 0$ (an integrable function of *t*) into state X which has an Erlang(*r*, *k*) distributed dwell time [with survival function $S_r^k$ from Eq. (7c)]. Let *x*(*t*) be the (mean field) amount in state X at time *t* and assume $x(0)=x_0$.

The mean field integral equation for this scenario (see Fig. 1a) is

14 $$\begin{aligned} x(t) =\; x_0S_r^k(t) + \int ^t_{0} {\mathcal {I}}(s)\,S_r^k(t-s) ds. \end{aligned}$$

State X can be partitioned (see Fig. 1b) into *k* sub-states X$_i$, $i=1,\ldots ,k$, where particles in X$_i$ are those awaiting the *i*th event as the next event under a homogeneous Poisson process with rate *r*. Let $x_i(t)$ be the amount in X$_i$ at time *t*, and $x(t) = \sum _{j=1}^{k} x_j(t)$. Equation (14) is equivalent to the mean field ODEs

15a $$\begin{aligned} \frac{d}{dt}{x_1}(t)&=\; {\mathcal {I}}(t) -r\, x_1(t) \end{aligned}$$

15b $$\begin{aligned} \frac{d}{dt}{x_j}(t)&=\; r\, x_{j-1}(t) - r\, x_j(t), \quad j=2,\ldots ,k \end{aligned}$$

with initial conditions $x_1(0)=x_0$, $x_j(0)=0$ for $j\ge 2$, and

16 $$\begin{aligned} x_j(t)= x_0\,\frac{1}{r}\,g_r^j(t) + \int ^t_{0} {\mathcal {I}}(s) \frac{1}{r}\,g_r^j(t-s)ds. \end{aligned}$$

#### Proof

Substituting Eq. (7c) into Eq. (14) and then substituting Eq. (16) yields

17 $$\begin{aligned} \begin{aligned} x(t)&=\; x_0\,S_r^k(t) + \int ^t_{0} {\mathcal {I}}(s)\,S_r^k(t-s) \,ds \\&=\; x_0\,\sum _{j=1}^{k} \frac{1}{r}\,g_r^j(t) + \int ^t_{0}{\mathcal {I}}(s)\,\sum _{j=1}^{k} \frac{1}{r}\,g_r^j(t-s) \,ds \\&= \sum _{j=1}^{k} \left( x_0\, \frac{1}{r}\,g_r^j(t) + \int ^t_{0} {\mathcal {I}}(s)\;\frac{1}{r}\,g_r^j(t-s) \,ds \right) = \sum _{j=1}^{k} x_j(t). \end{aligned} \end{aligned}$$

Differentiating Eq. (16) (for $j=1,\ldots ,k$) yields Eq. (15) as follows.

For $j=1$, Eq. (16) reduces to

18 $$\begin{aligned} x_1(t) = x_0 e^{-r\,t} + \int ^t_{0} {\mathcal {I}}(s) e^{-r(t-s)}ds. \end{aligned}$$

Differentiating $x_1(t)$ using the Leibniz integral rule and substituting (18) yields

19 $$\begin{aligned} \frac{d}{dt}{x_1}(t) =\; - r x_0 e^{-r\,t} - r\int ^t_{0} {\mathcal {I}}(s) e^{-r(t-s)}ds + {\mathcal {I}}(t) \;=\; {\mathcal {I}}(t) - r x_1(t). \end{aligned}$$

Similarly, for $j\ge 2$, Lemma 1 yields

20 $$\begin{aligned} \begin{aligned} \frac{d}{dt}{x_j}(t)&=\; x_0\,\frac{1}{r}\,\frac{d}{dt}g_r^j(t) + \int ^t_{0} {\mathcal {I}}(s) \frac{d}{dt} \left( \frac{1}{r}\,g_r^j(t-s)\right) \,ds \\&=\; x_0\,\left( g_r^{j-1}(t)-g_r^j(t)\right) + \int ^t_{0} {\mathcal {I}}(s) \left( g_r^{j-1}(t-s)-g_r^j(t-s)\right) \,ds \\&=\; r\,\bigg ( \frac{x_0}{r}\,g_r^{j-1}(t) + \int ^t_{0} {\mathcal {I}}(s) \frac{1}{r} g_r^{j-1}(t-s)\,ds \bigg ) -\; r\,\bigg ( \frac{x_0}{r}\,g_r^{j}(t)\\&\quad + \int ^t_{0} {\mathcal {I}}(s) \frac{1}{r} g_r^j(t-s)\,ds\bigg ) =\; r\,x_{j-1}(t) - r\,x_j(t). \end{aligned} \end{aligned}$$

$$\square $$

The X$_j$ dwell time distributions are exponential with rate *r*. To see why, let $\chi _i$(t) (where $1\le i \le k$) be the (mean field) number of particles in state X at time *t* that have not reached the *i*th event. Then

21 $$\begin{aligned} \chi _i(t) = x_0\,S_r^i(t) + \int _0^t {\mathcal {I}}(s)\,S_r^i(t-s)\,ds \end{aligned}$$

and by Eq. (7) we see from Eqs. (18) and (21) that $x_j(t) = \chi _j(t) - \chi _{j-1}(t)$. That is, particles in state X$_j$ are those for which the $(j-1)$th event has occurred, but not the *j*th event. Thus, by properties of Poisson processes the dwell time in state X$_j$ must exponential with rate *r*.

### Standard LCT

**Fig. 2:**
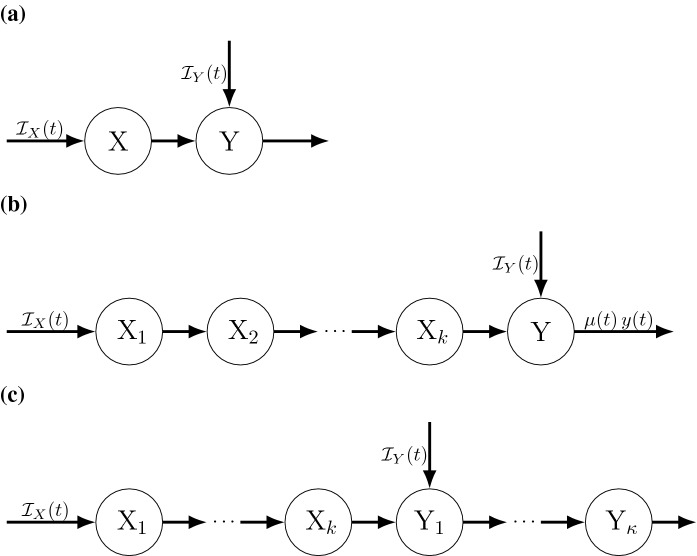
**(Standard LCT results)** This generic case assumes that the dwell times in state X (see panel **a**) are Erlang(*r*, *k*) distributed with inflow rates ${\mathcal {I}}_X(t)\ge 0$ into state X and ${\mathcal {I}}_Y(t)\ge 0$ into state Y. Panels **b** and **c** show sub-states resulting from applying the LCT and Corollary 1 assuming either (**b**) dwell times in state Y are determined by per-capita rate function $\mu (t)$, or (**c**) dwell times in Y follow and Erlang distribution with shape parameter $\kappa$

The following theorem and corollary together provide a more general, formal statement of the standard Linear Chain Trick (LCT) as used in practice. These extend Theorem 1 (compare Figs. 1, 2) to explicitly include that particles leaving X enter state Y then remain in Y according to an arbitrary distribution with survival function *S*, and include transitions into Y from other sources.

#### Theorem 2

(Standard LCT) Consider a continuous time dynamical system model of mass transitioning among various states, with inflow rate ${\mathcal {I}}_X(t)$ to a state X and an Erlang(*r*, *k*) distributed delay before entering state Y. Let *x*(*t*) and *y*(*t*) be the amount in each state, respectively, at time *t*. Further assume an inflow rate ${\mathcal {I}}_Y(t)$ into state Y from other non-X states, and that the underlying stochastic model assumes that the duration of time spent in state Y is determined by survival function $S_Y(t,\tau )$. Assume ${\mathcal {I}}_i(t)$ are integrable non-negative functions of *t*, and assume non-negative initial conditions $x(0)=x_0$ and $y(0)=y_0$.

The mean field integral equations for this scenario are

22a $$\begin{aligned} x(t)&=\; x_0\,S_r^k(t) + \int ^t_{0} {\mathcal {I}}_X(s)\,S_r^k(t-s) ds \end{aligned}$$

22b $$\begin{aligned} y(t)&=\; y_0S_Y(t,0) + \int ^t_{0} \bigg ({\mathcal {I}}_Y(\tau ) + x_0\,g_r^k(\tau ) \nonumber \\&\quad +\int ^\tau _0 {\mathcal {I}}_X(s)\,g^k_r(\tau -s)ds\bigg )S_Y(t,\tau )d\tau . \end{aligned}$$

Equations (22) are equivalent to

23a $$\begin{aligned} \frac{d}{dt}{x_1}(t)&=\; {\mathcal {I}}_X(t) -r x_1(t) \end{aligned}$$

23b $$\begin{aligned} \frac{d}{dt}{x_j}(t)&=\; r x_{j-1}(t) - r x_j(t), \quad j=2,\ldots ,k \end{aligned}$$

23c $$\begin{aligned} y(t)&=\; y_0S_Y(t,0) + \int ^t_{0} \underbrace{\left( {\mathcal {I}}_Y(\tau ) + r\,x_k(\tau )\right) }_{\text {Net input rate at time } \tau } S_Y(t,\tau )d\tau \end{aligned}$$

where $x(t) = \sum _{j=1}^{k} x_j(t)$, $x_1(0)=x_0$, $x_j(0)=0$ for $j\ge 2$, and

24 $$\begin{aligned} x_j(t)= x_0\,\frac{1}{r}\,g_r^j(t) + \int ^t_{0} {\mathcal {I}}_X(s) \frac{1}{r}\,g_r^j(t-s)ds. \end{aligned}$$

#### Proof

Eqs. (23a), (23b) and (24) follow from Theorem 1. Equation (23c) follows from substituting (24) into (22b). The definition of $x_j$ and initial condition $x(0)=x_0$ together imply $x_1(0)=x_0$ and $x_j(0)=0$ for the remaining $j\ge 2$. $\square$

#### Corollary 1

Integral equations like Eq. (23c) may have ODE representations depending on the Y dwell time distribution, i.e., $S_Y(t,\tau )$, for example:

1. If particles leave Y after a Poisson process 1st event time distributed dwell time [i.e., the per-capita loss rate from Y is $\mu (t)$], then $S_Y(t,\tau )=\exp (-\int _{\tau }^{t}\mu (u)\,du)$, and letting ${\mathcal {I}}(t)={\mathcal {I}}_Y(t)+rx_k(t)$, Theorem 2 yields
  25 $$\begin{aligned} \frac{d}{dt}{y}(t) =\; {\mathcal {I}}_Y(t) + r x_{k}(t) - \mu (t) y(t).\end{aligned}$$
2. If particles leave Y after an Erlang($\mu ,\kappa$) delay, then $S_Y(t,\tau )=S_\mu ^\kappa (t-\tau )$ and letting ${\mathcal {I}}(t)={\mathcal {I}}_Y(t)+rx_k(t)$ Theorem 2 gives that $y=\sum _{i=1}^\kappa y_i$ and
  26a $$\begin{aligned} \frac{d}{dt}{y_1}(t)&=\; {\mathcal {I}}_Y(t) + r x_{k}(t) -\mu \, y_1(t) \end{aligned}$$
  26b $$\begin{aligned} \frac{d}{dt}{y_i}(t)&=\; \mu \, y_{i-1}(t) - \mu \, y_i(t), \quad i=2,\ldots ,\kappa . \end{aligned}$$
3. As implied by 1 and 2 above, if the per-capita loss rate $\mu (t)=\mu$ is constant or time spent in Y is otherwise exponentially distributed, then
  27 $$\begin{aligned} \frac{d}{dt}{y}(t) =\; {\mathcal {I}}_Y(t) + r x_{k}(t) - \mu \, y(t).\end{aligned}$$
4. Any of the more general cases considered in the sections below.

#### Example 2

To illustrate how the Standard LCT can be used to substitute an implicit exponential dwell time distribution with an Erlang distribution, consider the SIR example discussed in the Introduction [Eqs. (2) and (3), see also Anderson and Watson 1980; Lloyd 2001a, b], but assume the dwell time distribution for the infected state I is Erlang (still with mean $1/\gamma$) with variance^6^$\sigma ^2$, i.e., by Eq. (6), Erlang with a rate $r=\gamma k$ and shape $k=\sigma ^2/\gamma ^2$.

By Theorem 2 and Corollary 1, with ${\mathcal {I}}_I(t)=\lambda (t)\,S(t)$ and $I(t)=\sum _{j=1}^k I_j(t)$, the corresponding mean field ODEs are

28a $$\begin{aligned} \frac{d}{dt}S(t)&=\; -\lambda (t)\,S(t) \end{aligned}$$

28b $$\begin{aligned} \frac{d}{dt}I_1(t)&=\; \lambda (t)\,S(t) - \gamma k\,I_1(t) \end{aligned}$$

28c $$\begin{aligned} \frac{d}{dt}I_j(t)&=\; \gamma k\,I_{j-1}(t) - \gamma k\,I_j(t), \quad \text { for } j=2,\ldots ,k \end{aligned}$$

28d $$\begin{aligned} \frac{d}{dt}R(t)&=\; \gamma k\,I_k(t). \end{aligned}$$

Notice that if $\sigma ^2=\gamma ^2$ (i.e., $k=1$), the dwell time in I is exponentially distributed with rate $\gamma$, $I(t)=I_1(t)$, and Eq. (28) reduce to Eq. (2).

This example nicely illustrates how using Theorem 2 to relax an exponential dwell time assumption implicit in a system of mean field ODEs is much more straightforward than constructing them after first deriving the integral equations, like Eq. (3), and then differentiating them using Lemma 1. In the sections below, we present similar theorems intended to be used for constructing mean field ODEs directly from stochastic model assumptions.

### Extended LCT for Poisson process *k*th event time distributed dwell times

The Standard LCT assumes an Erlang(*r*, *k*) distributed dwell time, i.e., a *k*th event time distribution under a homogeneous Poisson process with rate *r*. Here we generalize the Standard LCT by assuming the X dwell time follows a more general *k*th event time distribution under a Poisson process with rate *r*(*t*).

First, observe that Eq. (8) in Lemma 1 are more practical when written in terms of $\frac{1}{r} g^j_r(t)$ (see the proof of Theorem 1),

29a $$\begin{aligned} \frac{d}{dt}\bigg [\frac{1}{r} g^1_{r}(t)\bigg ]&=\; -\,r \bigg [\frac{1}{r} g^1_{r}(t)\bigg ], \end{aligned}$$

29b $$\begin{aligned} \frac{d}{dt}\bigg [\frac{1}{r} g^j_{r}(t)\bigg ]&=\; r\bigg [\frac{1}{r} g^{j-1}_{r}(t)-\frac{1}{r} g^{j}_{r}(t)\bigg ], \qquad j\ge 2 \end{aligned}$$

where $$\frac{1}{r} g^1_{r}(0)=1$$ and $$\frac{1}{r} g^j_{r}(0)=0$$.

#### Lemma 2

A similar relationship to Eq. (29) above (i.e., to Lemma 1) holds true for the Poisson process *j*th event time distribution density functions $h_{r}^j$ given by Eq. (9a). Specifically,

30a $$\begin{aligned} \frac{d}{dt}\bigg [\frac{1}{r(t)} h^1_{r}(t,\tau )\bigg ]&=\; -\,r(t) \bigg [\frac{1}{r(t)} h^1_{r}(t,\tau )\bigg ], \end{aligned}$$

30b $$\begin{aligned} \frac{d}{dt}\bigg [\frac{1}{r(t)} h^j_{r}(t,\tau )\bigg ]&=\; r(t)\bigg [\frac{1}{r(t)} h^{j-1}_{r}(t,\tau )-\frac{1}{r(t)} h^{j}_{r}(t,\tau )\bigg ], \end{aligned}$$

where $\frac{1}{r(\tau )}h^1_{r}(\tau ,\tau )=1$ and $\frac{1}{r(\tau )} h^j_{r}(\tau ,\tau )=0$ for $j\ge 2$. Note that, if for some *t*$r(t)=0$, this relationship can be written in terms of

31 $$\begin{aligned} u_r^k(t,\tau )\ \equiv \frac{m(t,\tau )^{k-1}}{(k-1)!}\,e^{-m(t,\tau )}, \end{aligned}$$

as shown in the proof below, where $h_r^k(t,\tau )=r(t)\,u_r^k(t,\tau )$, $u^1_{r}(\tau ,\tau )=1$, and $u^j_{r}(\tau ,\tau )=0$ for $j\ge 2$.

#### Proof

For $$j=1$$,

32 $$\begin{aligned} \frac{d}{dt}\bigg [u^1_{r}(t,\tau )\bigg ] =\; \frac{d}{dt}e^{-m(t,\tau )} =\; -r(t)\,e^{-m(t,\tau )} =\; -r(t)\,u^1_{r}(t,\tau ). \end{aligned}$$

Likewise, for $j\ge 2$, we have

33 $$\begin{aligned} \begin{aligned} \frac{d}{dt}\bigg [u^j_{r}(t,\tau )\bigg ]&=\; \frac{d}{dt} \frac{m(t,\tau )^{k-1}}{(k-1)!}\,e^{-m(t,\tau )} \\&=\; r(t)\, \frac{m(t,\tau )^{k-2}}{(k-2)!}\,e^{-m(t,\tau )} - r(t)\, \frac{m(t,\tau )^{k-1}}{(k-1)!}\,e^{-m(t,\tau )} \\&=\; r(t)\bigg [u^{j-1}_{r}(t,\tau )-u^{j}_{r}(t,\tau )\bigg ]. \end{aligned} \end{aligned}$$

$$\square $$

Lemma 2 allows us to generalize Erlang-based results like Theorem 2 to their time-varying counterparts with a time-dependent (or state-dependent) rate *r*(*t*), as in the following generalization of the Standard LCT (Theorem 2).

#### Theorem 3

(Extended LCT for dwell times distributed as Poisson process $k^\mathbf{th }$**event times)** Consider the Standard LCT in Theorem 2 but assume the X dwell time is a Poisson process *k*th event time, rate *r*(*t*). The corresponding mean field integral equations, where $h_r^j$ and ${\mathcal {S}}_r^j$ are given in Eq. (9), are

34a $$\begin{aligned} x(t)&=\; x_0\,{\mathcal {S}}_r^k(t,0) + \int ^t_{0} {\mathcal {I}}_X(s)\,{\mathcal {S}}_r^k(t,s) ds \end{aligned}$$

34b $$\begin{aligned} y(t)&=\; y_0S_Y(t,0) + \int ^t_{0} \bigg ({\mathcal {I}}_Y(\tau ) + x_0\,h_r^k(\tau ,0) \nonumber \\&\quad +\int ^\tau _0 {\mathcal {I}}_X(s)\,h^k_r(\tau ,s)ds\bigg )S_Y(t,\tau )d\tau . \end{aligned}$$

The above Eq. (34), with $x(t) = \sum _{j=1}^{k} x_j(t)$, are equivalent to

35a $$\begin{aligned} \frac{d}{dt}{x_1}(t)&=\; {\mathcal {I}}_X(t) -r(t)\, x_1(t) \end{aligned}$$

35b $$\begin{aligned} \frac{d}{dt}{x_j}(t)&=\; r(t)\, x_{j-1}(t) - r(t)\, x_j(t), \quad j=2,\ldots ,k \end{aligned}$$

35c $$\begin{aligned} y(t)&=\; y_0\,S_Y(t,0) + \int ^t_{0} \left( {\mathcal {I}}_Y(\tau ) + r(\tau )\,x_k(\tau )\right) S_Y(t,\tau )d\tau \end{aligned}$$

with initial conditions $x_1(0)=x_0$, $x_j(0)=0$ for $j\ge 2$ and

36 $$\begin{aligned} x_j(t)= x_0\,\frac{1}{r(t)}\,h_r^j(t,0) + \int ^t_{0} {\mathcal {I}}_X(s) \frac{1}{r(t)}\,h_r^j(t,s)ds. \end{aligned}$$

Equation (35c) may be further reduced to ODEs, e.g., via Corollary 1.

#### Proof

Substituting Eq. (9b) into Eq. (34a) and substituting Eq. (36) yields $x(t) = \sum _{j=1}^k x_j(t)$. Differentiating Eq. (36) with $j=1$ using the Liebniz integration rule as well as Eq. (30a) from Lemma 2 yields Eq. (35a). Likewise, for $j\ge 2$, differentiation of Eq. (36) and Lemma 2 yields

37 $$\begin{aligned} \begin{aligned} \frac{d}{dt}x_j(t)&=\; x(0)\,r(t)\,\bigg [ \frac{1}{r(t)} h^{j-1}_{r}(t,0) - \frac{1}{r(t)} h^{j}_{r}(t,0) \bigg ] \\&\quad + \int _0^t {\mathcal {I}}_X(s)\,r(t)\bigg [ \frac{1}{r(t)} h^{j-1}_{r}(t,\tau ) - \frac{1}{r(t)} h^{j}_{r}(t,\tau ) \bigg ] ds \\&=\; r(t) \big (x_{j-1}(t) - x_j(t)\big ). \end{aligned} \end{aligned}$$

Equation (35c) follows from substituting (36) into (34b). The definition of $x_j$ and initial condition $x(0)=x_0$ together imply $x_1(0)=x_0$ and $x_j(0)=0$ for the remaining $j\ge 2$. If $r(t)=0$ for some *t*, Eq. (35) still hold, since Eqs. (36) and (37) can be rewritten using *u* as in the proof of Lemma 2. $\square$

Having generalized the Standard LCT (Lemma 1 and Theorem 2) to include Poisson process *k*th event time distributed dwell times, we may now address more complex stochastic model assumptions and how they are reflected in the structure of corresponding mean field ODEs.

### Transitions to multiple states

**Fig. 3:**
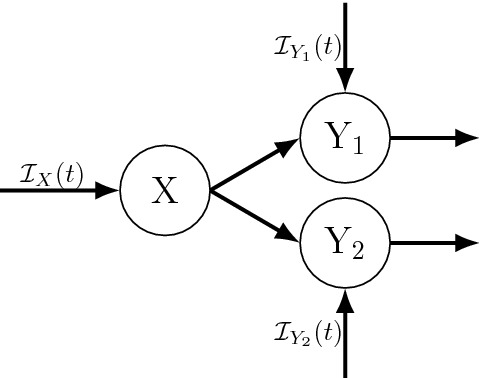
Example diagram of transitions out of a given state (X) and into multiple states (Y$_1$ and Y$_2$). Different assumptions about (1) the dwell times in X, and (2) rules governing the subsequent transitions to Y$_1$ and/or Y$_2$ will lead to different sub-state partitions of X, and thus different mean field equations, as detailed in Sect. 3.5. Fortunately, different scenarios often encountered in applications can be reduced to ODEs by applying the results in Sect. 3.5 as detailed in Theorems 4, 5, and 6 and as illustrated in Figs. 4, 5, and 6

Modeling the transition from one state to multiple states following a distributed delay (as illustrated in Fig. 3) can be done under different sets of assumptions about the underlying stochastic processes, particularly with respect to the rules governing how individuals are distributed across multiple recipient states upon exiting X, and how those rules depend on the dwell time distribution(s) for individuals in that state. Importantly, those different sets of assumptions can yield very different mean field models (e.g., see Feng et al. 2016) and so care must be taken to make those assumptions appropriately for a given application.

While modelers have some flexibility to choose appropriate assumptions, in practice modelers sometimes make unintended and undesirable implicit assumptions, especially when constructing ODE models using “rules of thumb” instead of deriving them from first principles. In this section we present results aimed at helping guide (a) the process of picking appropriate dwell time distribution assumptions, and (b) directly constructing corresponding systems of ODEs without deriving them from explicit stochastic models or intermediate integral equations.

Each of the three cases detailed below yield different mean field ODE models for the scenario depicted in Fig. 3.

First, in Sect. 3.5.1, we consider the extension of Theorem 3 where upon leaving X particles are distributed across $m\ge 1$ recipient states according to a generalized Bernoulli distribution with (potentially time varying) probabilities/proportions $p_{j}(t)$, $j=1,\ldots ,m$. Here the outcome of which state a particle transitions to is independent of the time spent in the first state.

Second, in Sects. 3.5.2 and 3.5.3, particles entering the first state (X) do not all follow the same dwell time distribution in X. Instead, upon entering X they are distributed across $n\ge 2$ sub-states of X, X$_i$, according to a generalized Bernoulli distribution, and each sub-state X$_i$ has a dwell time given by a Poisson process $k_i$th event time distribution with rate $r_i(t)$. That is, the X dwell time is a finite mixture of Poisson process event time distributions. Particles transition out of X into *m* subsequent states Y$_j$ according to the probabilities/proportions $p_{ij}(t)$, the probability of going to Y$_j$ from X$_i$, $i=1,\ldots ,n$ and $j=1,\ldots ,m$. Here the determination of which recipient state Y$_\ell$ a particle transitions to depends on which sub-state of X the particle was assigned to upon entering X (see Fig. 5).

Third, in Sect. 3.5.4, the outcome of which recipient state a particle transitions to upon leaving X is determined by a “race” between multiple competing Poisson process *k*th event time distributions, and is therefore not independent of the time spent in the first state (as in Sect. 3.5.1), nor is it pre-determined upon entry into X (as in Sects. 3.5.2 and 3.5.3). This result is obtained using yet another novel extension of Lemma 1 in which the dwell time in state X is the minimum of $n\ge 2$ independent Poisson process event time distributions.

Lastly (Sect. 3.5.5), we describe an equivalence between (1) the more complex case addressed in Sect. 3.5.4 assuming a dwell time that obeys the minimum of Poisson process 1st event times, before being distributed across *m* recipient states, and (2) the conceptually simpler case in Sect. 3.5.1 where the dwell time follows a single Poisson process 1st event time distribution before being distributed among *m* recipient states. This is key to understanding the scope of the Generalized Linear Chain Trick in Sect. 3.7.

#### Transition to multiple states independent of the X dwell time distribution

**Fig. 4:**
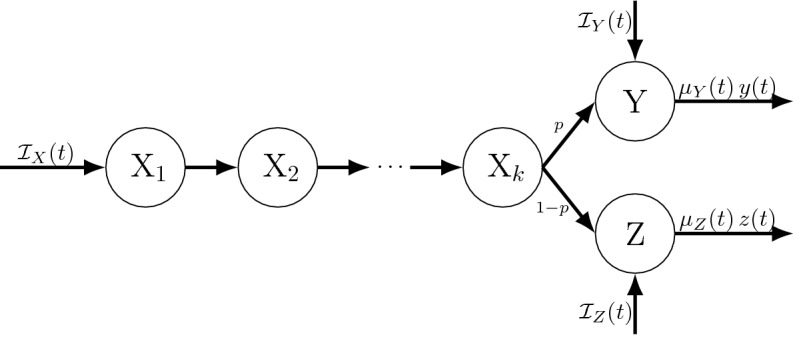
The special case of Fig. 3 under the assumptions of Theorem 4 (Extended LCT with proportional outputs to multiple states; see Sect. 3.5.1). Specifically, this case assumes that (1) the dwell time distribution for X is Erlang(*r*, *k*), and (2) upon exiting X particles are distributed to multiple recipient states, here Y and Z, with probabilities *p* and $1-p$, respectively

Here we extend the case in the previous section and assume that, upon leaving state X, particles can transition to one of *m* states (call them $Y_i$, $i=1,\ldots ,m$), and that a particle leaving X at time *t* enters state $Y_i$ with probability $p_i(t)$, where $\sum _{i=1}^m p_i(t)=1$ [i.e., particles are distributed across all Y$_i$ following a generalized Bernoulli distribution with parameter vector ${\mathbf {p}}(t)=(p_1(t),\ldots ,p_m(t))$]. See Fig. 4 for a simple example with constant ${\mathbf {p}}$ and $m=2$. An important assumption in this case is that the determination about which state a particle goes to after leaving X is made once it leaves X, and thus the state it transitions to upon exiting X is determined independent of the dwell time in X. Examples from the literature include Model II in Feng et al. (2016), where infected individuals (state X) either recovered (Y$_0$) or died (Y$_1$) after an Erlang distributed time delay.

##### Theorem 4

(Extended LCT with proportional output to multiple states) Consider the case addressed by Theorem 3, and further assume particles go to one of *m* states (call them Y$_j$) with $p_j(t)$ being the probability of going to Y$_j$. Let $S_j$ be the survival functions for the dwell times in Y$_j$.

**Fig. 5:**
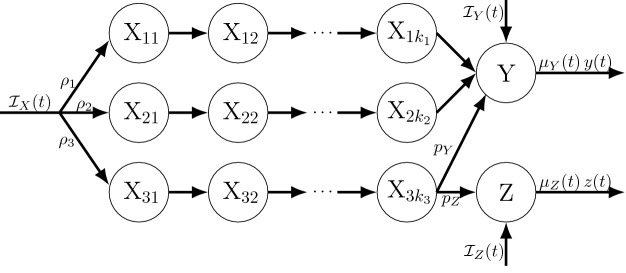
The sub-state diagram (cf. Fig. 3) for Example 4 after applying Theorem 5 (Extended LCT for finite mixtures of Poisson process event time distributions and output to multiple states). Upon entering X particles have an Erlang($r_i,k_i$) dwell time in X with probability $\rho _i$, $i=1,2,3$, i.e., the X dwell time follows an Erlang mixture distribution (see Sect. 3.5.3)

The mean field integral equations for this case, with $x(0)=x_0$ and $y_j(0)=y_{j0}$, are

38a $$\begin{aligned} x(t)&= x_0\,{\mathcal {S}}_{r}^{k}(t,0) + \int _0^t{\mathcal {I}}_X(s)\,{\mathcal {S}}_{r}^{k}(t,s)\,ds \end{aligned}$$

38b $$\begin{aligned} y_j(t)&= y_j(0)S_j(t,0) + \int _0^t\bigg ({\mathcal {I}}_j(\tau )+p_j(t)\bigg ( x_0\,h_{r}^{k}(\tau ,0) \nonumber \\&\quad + \int _0^\tau {\mathcal {I}}_X(s)h_{r}^{k}(\tau ,s)\,ds \bigg )\bigg )S_j(t,\tau ) d\tau \end{aligned}$$

These integral equations are equivalent to the following system of equations:

39a $$\begin{aligned} \frac{d}{dt}{x_1}(t)&=\; {\mathcal {I}}_X(t) -r(t)\, x_1(t) \end{aligned}$$

39b $$\begin{aligned} \frac{d}{dt}{x_i}(t)&=\; r(t)\, x_{i-1}(t) - r(t)\, x_i(t), \quad i=2,\ldots ,k \end{aligned}$$

39c $$\begin{aligned} y_j(t)&=\; y_j(0)S_j(t,0) + \int _0^t\bigg ({\mathcal {I}}_j(\tau )+p_j(t)\;r(t)\,x_k(\tau )\bigg )S_j(t,\tau ) d\tau \end{aligned}$$

where $x(t)=\sum _{i=1}^k x_i(t)$, $x_1(0)=x_0$, $x_i(0)=0$ for $i\ge 2$, and

40 $$\begin{aligned} x_i(t)= x_0 \frac{1}{r(t)} h_{r}^{k}(t,0) + \int _0^t{\mathcal {I}}_X(s) \frac{1}{r(t)} h_{r}^{k}(t,s)\,ds. \end{aligned}$$

Equations (39c) may be further reduced to ODEs, e.g., via Corollary 1.

##### Proof

Equations (39a), (39b) and (40) follow from Theorem 3. Equation (39c) follows from substitution of Eq. (40) into (38b). The derivation of Eq. (38b) is similar to the derivation in Appendix A.1 but accounts for the expected proportion entering each Y$_j$ at time *t* being equal to $p_j(t)$. $\square$

##### Example 3

Consider the example shown in Fig. 4, where the dwell time distribution for X is Erlang(*r*, *k*) and the dwell times in Y and Z follow 1st event times under nonhomogeneous Poisson processes with respective rates $\mu _Y(t)$ and $\mu _Z(t)$. By Theorem 4 the corresponding mean field ODEs are

41a $$\begin{aligned} \frac{d}{dt}{x_1}(t)&=\; {\mathcal {I}}_X(t) -r\, x_1(t) \end{aligned}$$

41b $$\begin{aligned} \frac{d}{dt}{x_i}(t)&=\; r\, x_{i-1}(t) - r\, x_i(t), \quad i=2,\ldots ,k \end{aligned}$$

41c $$\begin{aligned} \frac{d}{dt}{y}(t)&=\; {\mathcal {I}}_Y(t) + p\,r\, x_{k}(t) - \mu _Y(t) y(t) \end{aligned}$$

41d $$\begin{aligned} \frac{d}{dt}{z}(t)&=\; {\mathcal {I}}_Z(t) + (1-p)\,r\, x_{k}(t) - \mu _Z(t) z(t). \end{aligned}$$

#### Transition from sub-states of X with differing dwell time distributions and differing output distributions across states Y$_j$

In this second case, particles in state X can be treated as belonging to a heterogeneous population, where each remains in that state according to one of *N* possible dwell time distributions, the *i*th of these being the $k_i$th event time distribution under a Poisson process with rate $r_i(t)$. Each particle is assigned one of these *N* dwell time distributions (i.e., it is assigned to sub-state X$_i$) upon entry into X according to a generalized Bernoulli distribution with a (potentially time varying) probability vector $\varvec{\rho }(t)=(\rho _1(t),\ldots ,\rho _N(t))$, $\sum _{i=1}^N \rho _i(t) =1$. In contrast to the previous case, here the outcome of which recipient state a particle transitions to is not necessarily independent of the dwell time distribution.

Note that the dwell time distribution in this case is a finite mixture of *N* independent Poisson processes event time distributions. If a random variable *T* is a mixture of Erlang distributions, or more generally a mixture of *N* independent Poisson process event time distributions, then the corresponding density function (*f*) and survival function (${\varPhi }$) are

42a $$\begin{aligned} f_\theta (t,\tau )&=\; \sum _{i=1}^N \rho _i(\tau )\, h_{r_i}^{k_i}(t,\tau ) \end{aligned}$$

42b $$\begin{aligned} {\varPhi }_\theta (t,\tau )&=\; \sum _{i=1}^N \rho _i(\tau )\, {\mathcal {S}}_{r_i}^{k_i}(t,\tau ) =\; \sum _{i=1}^N \rho _i(\tau ) \sum _{j=1}^{k_i} \frac{1}{r_{i}(t)}\,h_{r_{i}}^j(t,\tau ) \end{aligned}$$

where $\varvec{\theta }(t)=(\rho _1(t), r_1(t), k_1, \ldots , \rho _N(t), r_N(t), k_N)$ is the potentially time varying parameter vector for the *N* distributions that constitute the mixture distribution. Note that if all $r_i(t)=r_i$ are constant, this is a mixture of Erlang distributions, or if also all $k_i=1$, a mixture of exponentials.

##### Theorem 5

(Extended LCT for dwell times given by mixtures of Poisson process event time distributions and outputs to multiple states) Consider a continuous time state transition model with inflow rate ${\mathcal {I}}_X(t)$ into state X. Assume that the duration of time spent in state X follows a finite mixture of *N* independent Poisson process event time distributions. That is, X can be partitioned into *N* sub-states X$_i$, $i=1,\ldots ,N$, each with dwell time distributions given by a Poisson process $k_i$th event time distributions with rates $r_i(t)$. Suppose the inflow to state X at time *t* is distributed among this partition according to a generalized Bernoulli distribution with probabilities $\rho _i(t)$, where $\sum _{i=1}^{N} \rho _i(t) = 1$, so that the input rate to X$_i$ is $\rho _i(t){\mathcal {I}}_X(t)$. Assume that particles leaving sub-state X$_i$ then transition to state Y$_\ell$ with probability $p_{i\ell }(t)$, $\ell =1,\ldots ,m$, where the duration of time spent in state Y$_\ell$ follows a delay distribution give by survival function $S_j$. Then we can partition each X$_i$ into X$_{ij}$, $j=1,\ldots ,k_i$, according to Theorem 3 and let *x*(*t*), $x_i(t)$, $x_{ij}(t)$, and $y_\ell (t)$ be the amounts in states X, X$_i$, X$_{ij}$, and Y$_\ell$ at time *t*, respectively. Assume non-negative initial conditions $x(0)=x_0$, $x_i(0)=\rho _i(0)x_0$, $x_{i1}(0)=\rho _i(0)\,x_0$, $x_{ij}(0)=0$ for $j\ge 2$, and $y_\ell (0)\ge 0$.

The mean field integral equations for this scenario are

43a $$\begin{aligned} x(t)&=\; x_0\,{\varPhi }_{\theta }(t,0) + \int ^t_{0} {\mathcal {I}}_X(s)\,{\varPhi }_{\theta }(t,s) ds \end{aligned}$$

43b $$\begin{aligned} y_\ell (t)&=\; y_\ell (0) S_\ell (t,0) + \int ^t_{0} \bigg ({\mathcal {I}}_\ell (\tau ) + \sum _{i=1}^N p_{ij}(\tau ) \bigg ( x_0\, \rho _i(\tau ) \,h_{r_i}^{k_i}(\tau ,0) \nonumber \\&\quad +\int ^\tau _0 \rho _i(s)\, {\mathcal {I}}_X(s)\,h_{r_i}^{k_i}(\tau , s) ds\bigg )\bigg ) S_\ell (t,\tau )d\tau . \end{aligned}$$

The above system of equations (43) are equivalent to

44a $$\begin{aligned} \frac{d}{dt}{x_{i1}}(t)&=\; \rho _i(t)\, {\mathcal {I}}_X(t) - r_i(t)\,x_{i1}(t), \quad i=1,\ldots ,N \end{aligned}$$

44b $$\begin{aligned} \frac{d}{dt}{x_{ij}}(t)&=\; r_i(t) \big (x_{i,j-1}(t) - x_{ij}(t)\big ), \quad i=1,\ldots ,N; \; j=2,\ldots ,k_i \end{aligned}$$

44c $$\begin{aligned} y_\ell (t)&=\; y_\ell (0) S_\ell (t,0) + \int ^t_{0} \bigg ({\mathcal {I}}_\ell (\tau ) \nonumber \\&\quad + \sum _{i=1}^N r_i(t)\,x_{ik_i}(\tau )\,p_{i\ell }(\tau ) \bigg ) S_\ell (t,\tau )d\tau \end{aligned}$$

with initial conditions $x_{i1}(0)=\rho _i(0)\,x_0$, $x_{ij}(0)=0$ for $j\ge 2$, where $x(t) = \sum _{i=1}^{N} x_i(t)$, and $x_i(t) = \sum _{j=1}^{k_i} x_{ij}(t)$. The amounts in X$_i$ and X$_{ij}$ are

45 $$\begin{aligned} x_i(t)&=\; \rho _i(0)\,x_0\,{\mathcal {S}}_{r_i}^{k_i}(t,0) + \int ^t_{0} \rho _i(s)\,{\mathcal {I}}_X(s)\,{\mathcal {S}}_{r_i}^{k_i}(t,s) ds \end{aligned}$$

46 $$\begin{aligned} x_{ij}(t)&=\; \rho _i(0)\,x_0 \frac{h_{r_i}^j(t,0)}{r_i(t)} + \int ^t_{0} \rho _i(s)\,{\mathcal {I}}_X(s)\;\frac{h_{r_i}^j(t,s)}{r_i(t)} \,ds. \end{aligned}$$

Equations (44c) may be reduced to ODEs, e.g., via Corollary 1.

##### Proof

Substituting Eq. (42b) into Eq. (43a) and then substituting Eq. (45) yields $x(t) = \sum _{i=1}^{N} x_{i}(t)$. Applying Theorem 3 to each X$_i$ [i.e., to each Eq. (45)] then yields Eqs. (46), (44a) and (44b). (Alternatively, one could prove this directly by differentiating Eq. (46) using Eq. (30) from Lemma 2). The $y_\ell (t)$ equations (44c) are obtained from (43b) by substitution of Eq. (46). $\square$

##### Example 4

Consider the scenario in Fig. 5, where particles entering state X at rate ${\mathcal {I}}_X(t)$ enter sub-state X$_i$ with probability $\rho _i$, $\rho _1+\rho _2+\rho _3=1$, and the X$_i$ dwell time is Erlang$(r_i,k_i)$ distributed. Particles exiting X$_1$ and X$_2$ transition to Y with probability 1, while particles exiting X$_3$ transition either to state Y or Z with probabilities $p_Y$ and $p_Z=1-p_Y$. Assume particle may also enter states Y and Z from sources other than state X (at rates ${\mathcal {I}}_Y(t)$ and ${\mathcal {I}}_Z(t)$, respectively), and the dwell times in those two states follow the 1st event times of independent nonhomogeneous Poisson processes with rates $\mu _Y(t)$ and $\mu _Z(t)$. Theorem 5 yields the following mean field ODEs (see Fig. 5).

47a $$\begin{aligned} \frac{d}{dt}{x_{i,1}}(t)&=\; \rho _i\, {\mathcal {I}}_X(t) - r_i x_{i,1}(t), \quad i=1,\ldots ,3, \end{aligned}$$

47b $$\begin{aligned} \frac{d}{dt}{x_{i,j}}(t)&=\; r_i \big (x_{i,j-1}(t) - x_{ij}(t)\big ), \quad j=2,\ldots ,k_i \end{aligned}$$

47c $$\begin{aligned} \frac{d}{dt}{y}(t)&=\; {\mathcal {I}}_Y(t) + r_1 \, x_{1,k_1}(t) + r_2\, x_{2,k_2}(t) \nonumber \\&\quad + r_3 \,p_Y\, x_{3,k_3}(t) - \mu _Y(t) y(t) \end{aligned}$$

47d $$\begin{aligned} \frac{d}{dt}{z}(t)&=\; {\mathcal {I}}_Z(t) + r_3\,p_Z\,x_{3,k_3}(t) - \mu _Z(t) z(t) . \end{aligned}$$

#### Extended LCT for dwell times given by finite mixtures of Poisson process event time distributions

It’s worth noting that in some applied contexts one may want to approximate a non-Erlang delay distribution with a mixture of Erlang distributions (see Sect. 3.7.1 and Appendix B for more details on making such approximations). Theorem 5 above details how assuming such a mixture distribution would be reflected in the structure of the corresponding mean field ODEs. This case can also be addressed in the more general context provided in Sect. 3.7.1.

#### Transition to multiple states following “competing” Poisson processes

We now consider the case where *T*, the time a particle spends in a given state X, follows the distribution given by $T=\min _i T_i$, the minimum of $n\ge 2$ independent random variables $T_i$, where $T_i$ has either an Erlang($r_i,k_i$) distribution or, more generally, a Poisson process $k_i$th event time distribution with rate $r_i(t)$. Upon leaving state X, particles have the possibility of transitioning to any of *m* recipient states $Y_\ell$, $\ell =1,\ldots ,m$, where the probability of transitioning to state Y$_\ell$ depends on which of the *n* random variables $T_i$ was the minimum. That is, if a particle leaves X at time $T=T_i=t$, then the probability of entering state Y$_\ell$ is $p_{i\ell (t)}$.

The distribution associated with *T* is not itself an Erlang distribution or a Poisson process event time distribution, however its survival function is the product^7^ of such survival functions, i.e.,

48 $$\begin{aligned} {\mathscr {S}}(t,\tau )\equiv \prod _{i=1}^{n}{\mathcal {S}}_{r_i}^{k_i}(t,\tau ). \end{aligned}$$

As detailed below, we can further generalize the recursion relation in Lemma 1 for the distributions just described above, which can then be used to produce a mean field system of ODEs based on appropriately partitioning X into sub-states.

Before considering this case in general, it is helpful to first describe the sub-states of X imposed by assuming the dwell time distribution described above, particularly the case where the distribution for each $T_i$ is based on 1st event times (i.e., all $k_i=1$). Recall that the minimum of *n* exponential random variables (which we may think of as 1st event times under a homogeneous Poisson process) is exponential with a rate that is the sum of the individual rates $r=\sum _{i=1}^n r_i$. More generally, it is true that the minimum of *n* 1st event times under independent Poisson processes with rates $r_i(t)$ is itself distributed as the 1st event time under a single Poisson processes with rate $r(t)\equiv \sum _{i=1}^n r_i(t)$, thus in this case ${\mathscr {S}}(t,\tau )= \prod _{i=0}^{n}{\mathcal {S}}_{r_i}^{1}(t,\tau )={\mathcal {S}}_{r}^{1}(t,\tau )$. Additionally, if particles leaving state X are then distributed across the recipient states Y$_\ell$ as described above, then this scenario is equivalent to the proportional outputs case described in Theorem 4 with a dwell time that follows a Poisson process 1st event time distribution with rate $r(t)\equiv \sum _{i=1}^n r_i(t)$ and a probability vector $p_\ell = \sum _{i=1}^n p_{i\ell }(t)r_i(t)/r(t)$, since $P(T=T_i)=r_i(T)/r(T)$. (This mean field equivalence of these two cases is detailed in Sect. 3.5.5.) Thus, the natural partitioning of X in this case is into sub-states with dwell times that follow *iid* 1st event time distributions with rate $r(t)\equiv \sum _{i=1}^{N} r_i(t)$.

We may now describe the mean field ODEs for the more general case using the following notation. To index the sub-states of X, consider the *i*th Poisson process and its $k_i$th event time distribution which defines the distribution of $T_i$. Let $a_i\in \{1,\ldots ,k_i\}$ denote the event number a particle is awaiting under the *i*th Poisson process. Then we can describe the particle’s progress through X according to its progress along each of these *n* Poisson processes using the index vector $\alpha \in {\mathcal {K}}$, where

49 $$\begin{aligned} {\mathcal {K}}=\{(a_1,a_2,\ldots ,a_n)\;|\;a_j\in \{1,\ldots ,k_j\}\}. \end{aligned}$$

Let ${\mathcal {K}}_{i}\subset {\mathcal {K}}$ denote the subset of indices where $a_i=k_i$ (where we think of particles in these sub-states as being poised to reach the $k_i$th event related to the *i*th Poisson process, and thus poised to transition out of state X).

To extend Lemma 2 for these distributions, define

50 $$\begin{aligned} u(t,\tau ,\alpha ) \equiv \prod _{i=1}^{n} e^{-m_i(t,\tau )} \frac{m_i(t,\tau )^{a_i-1}}{(a_i-1)!} \end{aligned}$$

where $m_i(t,\tau )=\exp \big (-\int _{\tau }^t r_i(s)ds\big )$, and $u(\tau ,\tau ,\alpha )=1$ if $\alpha =(1,\ldots ,1)$ and $u(\tau ,\tau ,\alpha )=0$ otherwise. Note that $\prod _{i=1}^{n} h_{r_i}^{a_i}(t,\tau ) = u(t,\tau ,\alpha )\prod _{i=1}^n r_i(t)$ (c.f. Lemma 2). Applying Eq. (9b) to ${\mathscr {S}}(t,\tau )$ in Eq. (48), the survival function given by Eq. (48) [c.f. Eq. (31) and (9b)] can be written

51 $$\begin{aligned} {\mathscr {S}}(t,\tau ) = \,\sum _{\alpha \in {\mathcal {K}}}u(t,\tau ,\alpha ). \end{aligned}$$

We will also refer to the quantities *u* and ${\mathscr {S}}$ with the *j*th element of each product (in *u*) removed using the notation

52a $$\begin{aligned} u_{{\setminus } j}(t,\tau ,\alpha )&\equiv \; \prod _{i=1, i\ne j}^n e^{-m_i(t,\tau )} \frac{m_i(t,\tau )^{a_i-1}}{(a_i-1)!} \end{aligned}$$

52b $$\begin{aligned} {\mathscr {S}}_{{\setminus } j}(t,\tau )&\equiv \; \sum _{\alpha \in {\mathcal {K}}_j} u_{{\setminus } j}(t,\tau ,\alpha ). \end{aligned}$$

This brings us to the following lemma, which generalizes Lemma 1 and Lemma 2 to distributions that are the minimum of *n* different (independent) Poisson process event times. As with the above lemmas, Lemma 3 will allow one to partition X into sub-states corresponding to each of the event indices in ${\mathcal {K}}$ describing the various stages of progress along each Poisson process prior to the first of them reaching the target event number.

##### Lemma 3

For *u* as defined in Eq. (50), differentiation with respect to *t* yields

53 $$\begin{aligned} \frac{d}{dt}u(t,\tau ,\alpha ) = \; \sum _{j=1}^n r_j(t)\,u(t,\tau ,\alpha _{j,-1})\,\mathbb {1}_{[a_j>1]}(\alpha ) - \sum _{j=1}^n r_j(t) u(t,\tau ,\alpha ) \end{aligned}$$

where the notation $\alpha _{j,-1}$ denotes the index vector generated by decrementing the *j*th element of $\alpha$, $a_j$ [assuming $a_j>1$; for example, $\alpha _{2,-1}=(a_1,a_2-1,\ldots ,a_n)$], and the indicator function $\mathbb {1}_{[a_j>1]}(\alpha )$ is 1 if $a_j>1$ and 0 otherwise.

##### Proof

Using the definition of *u* in Eq. (50) above, it follows that

54 $$\begin{aligned} \begin{aligned}&\frac{d}{dt}u(t,\tau ,\alpha ) =\; \frac{d}{dt}\prod _{i=1}^{n} e^{-m_i(t,\tau )} \frac{m_i(t,\tau )^{a_i-1}}{(a_i-1)!}\\&\quad =\sum _{j=1}^n\bigg (\prod _{\begin{array}{c} i=1\\ i\ne j \end{array}}^n e^{-m_i(t,\tau )} \frac{m_i(t,\tau )^{a_i-1}}{(a_i-1)!}\bigg )\bigg [-r_j(t)e^{-m_j(t,\tau )} \frac{m_j(t,\tau )^{a_j-1}}{(a_j-1)!} \\&\qquad + \mathbb {1}_{[a_j>1]}(\alpha )\,r_j(t)\,e^{-m_j(t,\tau )}\frac{m_j(t,\tau )^{a_j-2}}{(a_j-2)!}\bigg ] \\&\quad =\sum _{j=1}^n -r_j(t)\,\prod _{i=1}^{n} e^{-m_i(t,\tau )} \frac{m_i(t,\tau )^{a_i-1}}{(a_i-1)!} \; \\&\qquad + \sum _{j=1}^n \mathbb {1}_{[a_j>1]}(\alpha )\,r_j(t)\,e^{-m_j(t,\tau )}\frac{m_j(t,\tau )^{a_j-2}}{(a_j-2)!} \prod _{\begin{array}{c} i=1\\ i\ne j \end{array}}^n e^{-m_i(t,\tau )} \frac{m_i(t,\tau )^{a_i-1}}{(a_i-1)!} \\&\quad = \sum _{j=1}^n r_j(t)\,u(t,\tau ,\alpha _{j,-1})\,\mathbb {1}_{[a_j>1]}(\alpha ) - \sum _{j=1}^n r_j(t) u(t,\tau ,\alpha ). \end{aligned} \end{aligned}$$

$$\square $$

The next theorem details the LCT extension that follows from Lemma 3.

##### Theorem 6

(Extended LCT for dwell times given by competing Poisson processes) Consider a continuous time dynamical system model of mass transitioning among multiple states, with inflow rate ${\mathcal {I}}_X(t)$ to a state X. The distribution of time spent in state X (call it *T*) is the minimum of *n* random variables, i.e., $T=\min _{i}(T_i)$, $i=1,\ldots ,n$, where $T_i$ are either Erlang($r_i,k_i$) distributed or follow the more general (nonhomogeneous) Poisson process $k_i$th event time distribution with rate $r_i(t)$. Assume particles leaving X can enter one of *m* states Y$_\ell$, $\ell =1,\ldots ,m$. If a particle leaves X at time $T_i$ (i.e., $T_i$ occurred first, so $T=T_i$), and then the particle transitions into state $Y_\ell$ with probability $p_{i\ell }(T)$. Let *x*(*t*), and $y_\ell (t)$ be the amount in each state, respectively, at time *t*, and assume non-negative initial conditions.

The mean field integral equations for this scenario, for $\ell =1,\ldots ,m$ and $i=1,\ldots ,n$, are

55a $$\begin{aligned} x(t)&=\; x_0\,{\mathscr {S}}(t,0) + \int ^t_{0} {\mathcal {I}}_X(s)\,{\mathscr {S}}(t,s)ds \end{aligned}$$

55b $$\begin{aligned} y_\ell (t)&=\; y_{\ell }(0)S_\ell (t,0) + \int ^t_{0} \bigg ({\mathcal {I}}_\ell (\tau ) + \sum _{i=1}^n p_{i\ell } \bigg ( x_0\,{\mathscr {S}}_{{\setminus } i}(\tau ,0)\,h_{r_i}^{k_i}(\tau ,0) \; \nonumber \\&\quad + \int _{0}^{\tau } {\mathcal {I}}_X(s) {\mathscr {S}}_{{\setminus } i}(\tau ,s)\,h_{r_i}^{k_i}(\tau ,s) ds\bigg )\bigg )S_\ell (t,\tau )d\tau . \end{aligned}$$

Equations (55) above are equivalent to

56a $$\begin{aligned} \frac{d}{dt}x_{(1,\ldots ,1)}(t)&=\; {\mathcal {I}}_X(t) - r(t)\,x_{(1,\ldots ,1)}(t), \end{aligned}$$

56b $$\begin{aligned} \frac{d}{dt}x_\alpha (t)&=\;\sum _{i=1}^{n} r_i(t)\,x_{\alpha _{i,-1}}(t)\,\mathbb {1}_{[a_i>1]}(\alpha ) \;-\; r(t) \, x_{\alpha }(t) \end{aligned}$$

56c $$\begin{aligned} y_\ell (t)&=\; y_{\ell }(0) S_\ell (t,0) \nonumber \\&\quad + \int ^t_{0} \bigg ({\mathcal {I}}_\ell (\tau ) + \sum _{i=1}^n p_{i\ell }(\tau )\sum _{\alpha \in {\mathcal {K}}_i} r_i(t)\,x_\alpha (\tau ) \bigg )S_\ell (t,\tau )d\tau \end{aligned}$$

for all $\alpha \in {\mathcal {K}}{\setminus }(1,\ldots ,1)$, $r(t)=\sum _{i=1}^{n}r_i(t)$, $x(t)=\sum _{\alpha \in {\mathcal {K}}} x_\alpha (t)$, and

57 $$\begin{aligned} x_\alpha (t) = x_0\,u(t,0,\alpha ) + \int _0^t{\mathcal {I}}_X(s)\,u(t,s,\alpha )\,ds. \end{aligned}$$

The $y_\ell (t)$ equations (56c) may be further reduced to a system of ODEs, e.g., via Corollary 1.

##### Proof

Substituting Eq. (51) into Eq. (55a) yields

58 $$\begin{aligned} \begin{aligned} x(t)&= x_0\,\sum _{\alpha \in {\mathcal {K}}} u(t,0,\alpha ) + \int ^t_{0} {\mathcal {I}}_X(s)\,\sum _{\alpha \in {\mathcal {K}}}u(t,s,\alpha )ds \\&= \sum _{\alpha \in {\mathcal {K}}} \bigg ( x_0\, u(t,0,\alpha ) + \int ^t_{0} {\mathcal {I}}_X(s)\, u(t,s,\alpha )ds \bigg ) = \sum _{\alpha \in {\mathcal {K}}} x_\alpha (t). \end{aligned} \end{aligned}$$

Differentiating (57) yields equations Eqs. (56a) and (56b) as follows. First, if $\alpha =(1,\ldots ,1)$ then by Lemma 3

59 $$\begin{aligned} \begin{aligned} \frac{d}{dt}{x_{(1,\ldots ,1)}}(t)&=\; -x_0\,\sum _{i=1}^n r_i(t)\,u(t,0,\alpha ) \\&\quad - \sum _{i=1}^n r_i(t)\,\int ^t_{0} {\mathcal {I}}_X(s)\, u(t,s,\alpha )ds + {\mathcal {I}}_X(t) \\&=\; {\mathcal {I}}_X(t) - \sum _{i=1}^n r_i(t)\, x_{(1,\ldots ,1)}(t). \end{aligned} \end{aligned}$$

Next, if $\alpha$ has any $a_i>1$, differentiating Eq. (57) and applying Lemma 3 yields

60 $$\begin{aligned} \begin{aligned}&\frac{d}{dt}{x_\alpha }(t) =\; x_0\, \frac{d}{dt}u(t,0,\alpha ) + \int ^t_{0} {\mathcal {I}}_X(s)\; \frac{d}{dt}u(t,s,\alpha ) \,ds \\&\quad =\; x_0\, \bigg ( \sum _{i=1}^{n} r_i(t)u(t,0,\alpha _{i,-1})\,\mathbb {1}_{[a_i>1]}(\alpha ) - \sum _{i=1}^{n}r_i(t)u(t,\alpha ) \bigg ) \; \\&\qquad + \int ^t_{0} {\mathcal {I}}_X(s)\bigg ( \sum _{i=1}^{n} r_i(t)\,u(t,s,\alpha _{i,-1})\,\mathbb {1}_{[a_i>1]}(\alpha ) - \sum _{i=1}^{n}r_i(t)\,u(t,s,\alpha )\bigg )\,ds \\&\quad =\; \sum _{i=1}^{n} r_i(t)\,x_{\alpha _{i,-1}}(t)\,\mathbb {1}_{[a_i>1]}(\alpha ) - \sum _{i=1}^{n} r_i(t)\,x_{\alpha }(t) \end{aligned} \end{aligned}$$

Note that, by the definitions of $x_\alpha$ and *u* that initial condition $x(0)=x_0$ becomes $x_{(1,\ldots ,1)}(0)=x_0$ and $x_\alpha (0)=0$ for the remaining $\alpha \in {\mathcal {K}}$.

Eqs. (55b) become (56c), where ${\mathcal {K}}_i=\{\alpha \;|\;\alpha \in {\mathcal {K}},\;a_i=k_i\}$, by substituting Eqs. (52), (57), and ${\mathscr {S}}_{{\setminus } i}(t,\tau )\,h_{r_i}^{k_i}(t,\tau ) = \sum _{\alpha \in {\mathcal {K}}_i} r_i(t)\,u(t,\tau ,\alpha )$, which yields

61 $$\begin{aligned} \begin{aligned}&x_0\,{\mathscr {S}}_{{\setminus } i}(\tau ,0)h_{r_i}^{k_i}(\tau ,0) + \int _{0}^{\tau } {\mathcal {I}}_X(s) {\mathscr {S}}_{{\setminus } i}(\tau ,s)h_{r_i}^{k_i}(\tau ,s) ds \; \\&\quad = x_0 \sum _{\alpha \in {\mathcal {K}}_i} r_i(t)\,u(\tau ,0,\alpha ) + \int _{0}^{\tau } {\mathcal {I}}_X(s) \sum _{\alpha \in {\mathcal {K}}_i} r_i(t)\,u(\tau ,s,\alpha ) ds \; \\&\quad = r_i(t) \sum _{\alpha \in {\mathcal {K}}_i} \bigg (x_0\, u(\tau ,0,\alpha ) + \int _{0}^{\tau } {\mathcal {I}}_X(s) u(\tau ,s,\alpha ) ds \bigg ) =\; \sum _{\alpha \in {\mathcal {K}}_i}r_i(t) \, x_\alpha (\tau ). \\ \end{aligned} \end{aligned}$$

$$\square $$

**Fig. 6:**
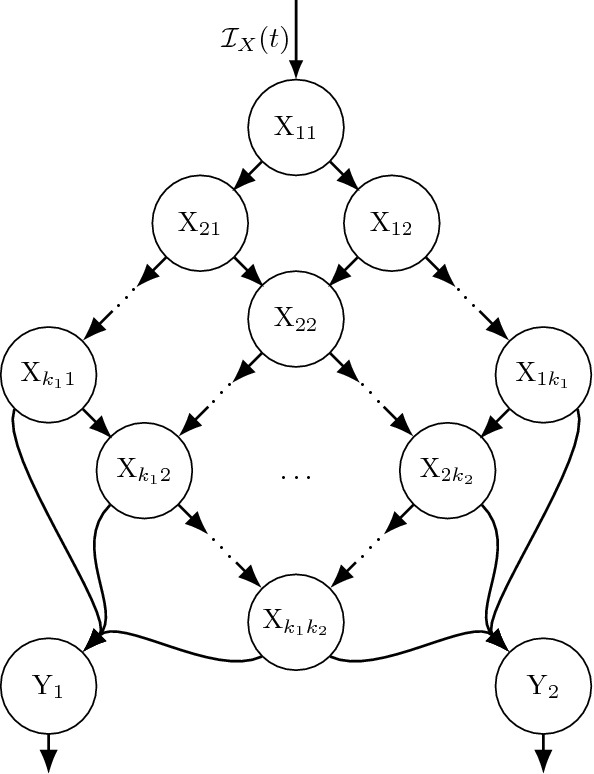
The sub-state diagram (cf. Fig. 3) for the scenario detailed in Example 5 after the application of Theorem 6 (extended LCT for dwell times given by competing Poisson processes). Here the X dwell time distribution is the minimum of two Erlang random variables $T_i\sim$Erlang($r_i,k_i$), $i=1,2$, which can be thought of as event time distribution under two homogeneous Poisson processes as detailed in the main text. We here assume that whichever of these occurs first determines whether particles leaving X transition to Y$_1$ or Y$_2$, respectively

##### Example 5

See Fig. 6. Suppose the X dwell time is $T=\min (T_1,T_2)$ where $T_1$ and $T_2$ are the $k_1$th and $k_2$th event time distributions under independent Poisson processes (call these PP1 and PP2) with rates $r_1(t)$ and $r_2(t)$, respectively. Assume that, upon leaving X, particles transition to Y$_1$ if $T=T_1$ or to Y$_2$ if $T=T_2$. By Theorem 6, we can partition X into sub-states defined by which event (under each Poisson process) particles are awaiting next. Upon entry into X, all particles enter sub-state X$_{1,1}$ where they each await the 1st events under PP1 or PP2. If the next event to occur for a given particle is from PP1, the particle transitions to X$_{2,1}$ where it awaits a 2nd event from PP1 or 1st event from PP2 (hence the subscript notation). Likewise, if PP2’s 1st event occurs before PP1’s 1st event, the particle would transition to X$_{1,2}$, and so on. Particles would leave these two states to either X$_{2,2}$, Y$_1$, or Y$_2$ depending on which event occurs next. Under these assumptions, with $k_1=k_2=2$ and exponential Y$_i$ dwell times with rates $\mu$, then the corresponding mean field equations (using $r(t) = r_1(t)+r_2(t)$) are

62a $$\begin{aligned} \frac{dx_{11}}{dt}&=\; {\mathcal {I}}_X(t) - r(t)\,x_{11}(t) \end{aligned}$$

62b $$\begin{aligned} \frac{dx_{21}}{dt}&=\; r_1(t)\,x_{11}(t) - r(t)\,x_{21}(t) \end{aligned}$$

62c $$\begin{aligned} \frac{dx_{12}}{dt}&=\; r_2(t)\,x_{11}(t) - r(t)\,x_{12}(t) \end{aligned}$$

62d $$\begin{aligned} \frac{dx_{22}}{dt}&=\; r_1(t)\,x_{12}(t) + r_2(t)\,x_{21}(t) - r(t)\,x_{12}(t) \end{aligned}$$

62e $$\begin{aligned} \frac{dy_{1}}{dt}&=\; r_1(t)\,x_{22}(t) - \mu (t)\,y_{1}(t) \end{aligned}$$

62f $$\begin{aligned} \frac{dy_{2}}{dt}&=\; r_2(t)\,x_{22}(t) - \mu (t)\,y_{2}(t). \end{aligned}$$

It’s worth pointing out that the dwell times for the above sub-states of X are all identically distributed Poisson process 1st event times (note the loss rates in Eqs. (62a)–(62d), and recall the weak memorylessness property from Sect. 3.1.2). All particles in a X sub-state at time $\tau$ will spend a remaining amount of time in that state that follows a 1st event time distributions under a Poisson process with rate $r(t)=r_1(t)+r_2(t)$. This is a slight generalization of the familiar fact that the minimum of *n* independent exponentially distributed random variables (with respective rates $r_i$) is itself an exponential random variable (with rate $r\equiv \sum _{i=1}^n r_i$). The next section addresses the generality of this observation about the sub-states of X.

#### Mean field equivalence of proportional outputs and competing Poisson processes for 1st event time distributions

The scenarios described in Sect. 3.5.1 (proportional distribution across multiple states $Y_\ell$ after an Erlang dwell time in X) and Sect. 3.5.4 (proportional distribution across multiple states based upon competing Poisson processes), can lead to equivalent mean field equations when the X dwell times follow Poisson process 1st event time distributions, as is Example 5. This equivalence is detailed in Theorem 7, and is an important aspect of the GLCT detailed in Sect. 3.7 because it helps to show how sub-states with dwell times distributed as Poisson process 1st event times are the fundamental buildings blocks of the GLCT.

##### Theorem 7

(Mean field equivalence of proportional outputs and competing Poisson processes for 1st event time distributions) Consider the special case of Theorem 6 (the Extended LCT for competing Poisson processes) where X has a dwell time given by $T=\min _i T_i$, where each $T_i$ is a Poisson process 1st event time with rate $r_i(t)$, $i=1,\ldots ,n$ and particles transition to Y$_\ell$ with probability $p_{i\ell }(T)$ when $T=T_i$. The corresponding mean field model is equivalent to the special case of Theorem 4 (the Extended LCT for multiple outputs) where the X dwell time is a Poisson process 1st event time distribution with rate $r(t)=\sum _{i=1}^n r_i(t)$, and the transition probability vector for leaving X and entering state Y$_\ell$ is given by $p_\ell (t)=\sum _{i=1}^n p_{i\ell }(t)\,r_i(t)/r(t)$.

##### Proof

First, in this case ${\mathscr {S}}(t,\tau ) = \prod _{i=0}^{n}{\mathcal {S}}_{r_i}^{1}(t,\tau )={\mathcal {S}}_{r}^{1}(t,\tau )$. Since all $k_i=1$, the probability that $T=T_i$ is $r_i(T)/r(T)$, thus the probability that a particle leaving X at *t* goes to Y$_\ell$ is $p_\ell (t)=\sum _{i=1}^n \frac{r_i(t)}{r(t)}p_{i\ell }(t)$. Substituting the above equalities into the mean field Eq. (56a) (where there’s only one possible index in ${\mathcal {K}}=\{(1,1,\ldots ,1)\}$) and (56c) gives

63a $$\begin{aligned} \frac{d}{dt}x(t)&=\; {\mathcal {I}}_X(t) - r(t)\,x(t) \end{aligned}$$

63b $$\begin{aligned} y_j(t)&=\; y_{j}(0)S_j(t,0)\; + \int ^t_{0} \bigg ({\mathcal {I}}_j(\tau ) + r(t)\,p_j(\tau )\,x(\tau )\bigg ) S_j(t,\tau ) d\tau \end{aligned}$$

which are the mean field equations for the aforementioned special case of Theorem 4. $\square$

### Modeling intermediate state transitions: reset the clock, or not?

**Fig. 7:**
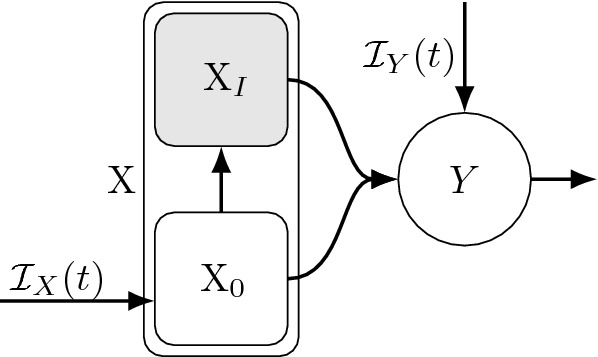
Should the overall dwell time distribution for state X be “reset” by the transition from base sub-state X$_0$ to intermediate sub-state X$_I$ (i.e., should the dwell time in state X$_I$ be independent of the time already spent in X$_0$?), or should the X$_I$ dwell time be conditioned on time already spent in X$_0$ so that the X$_0\rightarrow$X$_I$ transition does not alter the overall dwell time in state X? How do these different assumptions alter the structure of the corresponding mean field ODEs? We answer these question in Sect. 3.6 where we describe how to apply the LCT in scenarios with intermediate states, assuming in Sect. 3.6.1 that the dwell time distribution for X$_I$ is independent of the amount of time spent in X$_0$, and assuming in Sect. 3.6.2 that the overall dwell time for X is unaffected by transitions from X$_0$ to X$_I$

We next describe how to apply extensions of the LCT in two similar but distinctly different scenarios (see Fig. 7) where the transition to one or more intermediate sub-states either resets an individual’s overall dwell time in state X (by assuming the time spent in an intermediate sub-state X$_{I_i}$ is independent of time already spent in X$_0$; see Sect. 3.6.1), or instead leaves the overall dwell time distribution for X unchanged (by conditioning the time spent in intermediate state X$_{I_i}$ on the time already spent in X$_0$; see Sect. 3.6.2).

An example of these different assumptions leading to important differences in practice comes from Feng et al. (2016) where individuals infected with Ebola can either leave the infected state (X) directly (either to a recovery or death), or after first transitioning to an intermediate hospitalized state (X$_I$) which needs to be explicitly modeled in order to incorporate a quarantine effect into the rates of disease transmission (i.e., the force of infection should depend on the number of non-quarantined individuals, i.e., X$_0$). As shown in Feng et al. (2016), the epidemic model output depends strongly upon whether or not it is assumed that moving into the hospitalized sub-state impacts the distribution of time spent in the infected state X.

To most simply illustrate these two scenarios, consider the simple case in Fig. 7 where a single intermediate sub-state $X_I$ is being modeled, and particles enter X into sub-state X$_0$ at rate ${\mathcal {I}}_X(t)$. Let X$=$X$_0\cup$X$_I$. Both cases assume particles transition out of X$_0$ either to sub-state X$_I$ or they leave state X directly and enter state Y. Both cases also assume the distribution of time spent in X$_0$ is $T_*=$min($T_0,T_1$) where particles transition to X$_I$ if $T_1<T_0$ (i.e., if $T=T_1$) or to Y if $T_0<T_1$ (where each $T_i$ is the $k_i$th event time under Poisson processes with rates $r_0(t)$ and $r_1(t)$ (see Sects. 3.5.4 and 3.5.5). Let $T_I$ denote the distribution of time spent in intermediate state X$_I$. The first case assumes $T_I$ is independent of time spent in X$_0$ (i.e., the transition to X$_I$ ‘resets the clock’; see Sect. 3.6.1). The second case assumes $T_I$ is conditional on time already spent in X$_0$ (call it $t_0$), such that the total amount of time spent in X, $t_0+T_I$, is equivalent in distribution to $T_0$ (i.e., the transition to X$_I$ does not change the overall distribution of time spent in X; see Sect. 3.6.2).

In the next two sections, we provide extensions of the LCT for generalizations of these two scenarios, extended to multiple possible intermediate states with eventual transitions out of X into multiple recipient states.

#### Intermediate states that reset dwell time distributions

First, we consider the case in which the time spent in the intermediate state X$_I$ is independent of the time already spent in X (i.e., in the base state X$_0$). This is arguably the more commonly encountered (implicit) assumption found in ODE models that aren’t explicitly derived from a stochastic model and/or mean field integro-differential delay equations.

The construction of mean field ODEs for this case is a straightforward application of Theorem 6 from the previous section, combined with the extended LCT with output to multiple states (Theorem 4). Here we have extended this scenario to include $M_X$ intermediate sub-states X$_{I_j}$ where the transition to those sub-states from base state X$_0$ is based on the outcome of *N* competing Poisson process event time distributions ($T_i$), and upon leaving the intermediate states particles transition out of state X into one of $M_Y$ possible recipient states Y$_\ell$.

##### Theorem 8

(Extended LCT with dwell time altering intermediate sub-state transitions) Suppose particles enter X at rate ${\mathcal {I}}_X(t)$ into a base sub-state X$_0$. Assume particles remain in X$_0$ according to a dwell time distribution given by *T*, the minimum of $N+1$ independent Poisson process $k_i$th event time distributions with rates $r_i(t)$, $i=0,\ldots ,N$, i.e., $T=\min _i(T_i)$. Particles leaving X$_0$ transition to one of $M_X\ge 1$ intermediate sub-states X$_{I_i}$ or to one of $M_Y\ge 1$ recipient states $Y_\ell$ according to which $T_i=T$. If $T_0=T$ then the particle leaves X and the probability of transitioning to Y$_\ell$ is $p_{0\ell }(T)$, where $\sum _{\ell =1}^{M_Y} p_{0\ell }(T)=1$. If $T_i=T$ for $i\ge 1$ then the particle transitions to X$_{I_j}$ with probability $p_{ij}(T)$, where $\sum _{j=1}^{M_X} p_{ij}(T)=1$. Particles in intermediate state $X_{I_j}$ remain there according to the $\kappa _i$th event times under a Poisson process with rate $\varrho _i(t)$, and then transition to state Y$_\ell$ with probability $q_{j\ell }(t)$, where (for fixed *t*) $\sum _{\ell =1}^{M_Y} q_{j\ell }(t)=1$, and they remain in Y$_\ell$ according to a dwell time with survival function $S_\ell (t,\tau )$.

In this case the corresponding mean field equations are

64a $$\begin{aligned} \frac{d}{dt}x_{0(1,\ldots ,1)}(t)&=\; {\mathcal {I}}_X(t) - \sum _{i=0}^N r_i(t) \,x_{(1,\ldots ,1)}(t) \end{aligned}$$

64b $$\begin{aligned} \frac{d}{dt}x_{0\alpha }(t)&=\; \sum _{i=0}^{N} r_i(t) \bigg ( x_{0\alpha _{i,-1}}(t)\,\mathbb {1}_{[a_i>1]}(\alpha ) - x_{0\alpha }(t)\bigg ) \end{aligned}$$

64c $$\begin{aligned} \frac{d}{dt}x_{I_{j1}}(t)&=\; {\mathcal {I}}_{X_{Ij}}(t) + p_{ij}(t)\bigg (\sum _{\alpha \in {\mathcal {K}}_i} r_i(t)\,x_{0\alpha }(t)\bigg ) - \varrho _j(t)\,x_{I_{j1}}(t) \end{aligned}$$

64d $$\begin{aligned} \frac{d}{dt}x_{I_{jk}}(t)&=\; \varrho _j(t)\big (x_{I_{j,k-1}}(t) - x_{I_{jk}}(t)\big ), \;\qquad k=2,\ldots ,\kappa _j \end{aligned}$$

64e $$\begin{aligned} y_\ell (t)&=\; y_{\ell }(0)\,S_\ell (t,0) + \int ^t_{0} \bigg ({\mathcal {I}}_{Y_\ell }(\tau ) + p_{0\ell }(\tau ) \sum _{\alpha \in {\mathcal {K}}_0} r_0(\tau )\,x_{0\alpha }(\tau ) \nonumber \\&\quad + \sum _{j=1}^{M_X} \varrho _j(\tau )\,x_{I_{j\kappa _j}}(\tau )\, q_{j\ell }(\tau ) \bigg ) S_Y(t,\tau )\,d\tau . \end{aligned}$$

where the amount in base sub-state X$_0$ is $x_0(t)=\sum _{\alpha \in {\mathcal {K}}}x_{0\alpha }(t)$, the amount in the *j*th intermediate state X$_{I_j}$ is $x_{Ij}(t)=\sum _{k=1}^{\kappa _j} x_{I_{jk}}(t)$ (see Theorem 6 for notation), ${\mathcal {K}}=\{(a_0,a_1,\ldots ,a_N)\;|\;a_j\in \{1,\ldots ,k_j\}\}$, ${\mathcal {K}}_i=\{\alpha \in {\mathcal {K}} | a_i=k_i\}$, $j=1,\ldots ,N$, $\ell = 1,\ldots ,M_Y$, and in Eq. (64b) $\alpha =(a_0,\ldots ,a_N) \in {\mathcal {K}}{\setminus }(1,\ldots ,1)$. Note that the $y_\ell (t)$ equations (64e) may be further reduced to a system of ODEs, e.g., via Corollary 1, and that more complicated distributions for dwell times in intermediate states X$_{I_i}$ (e.g., an Erlang mixture) could be similarly modeled according to other cases addressed in this manuscript.

##### Proof

This follows from applying Theorem 6 to X$_0$ and treating the intermediate states X$_{I_j}$ as recipient states, then applying Theorem 4 to each intermediate state to partition each X$_{I_j}$ into X$_{I_{jk}}$, $k=1,\ldots ,\kappa _j$, yielding Eq. (64). $\square$

##### Example 6

**Fig. 8:**
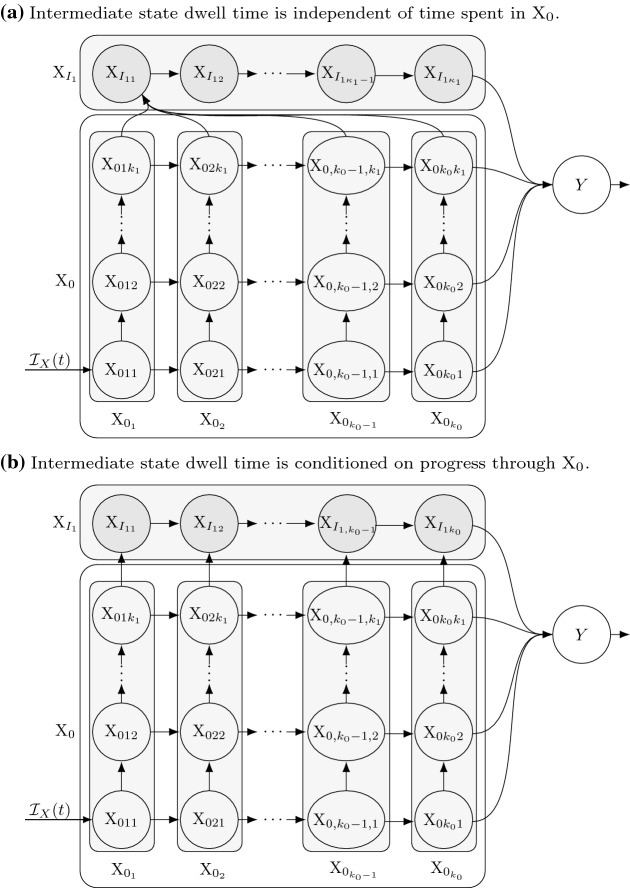
When modeling sub-state transitions, e.g., from a base state X$_0$ to intermediate state X$_{I_1}$ (state X$=$X$_0\cup$X$_{I_1}$), the intermediate sub-state dwell-time can be **(a)** independent of time already spent in X$_0$ (Sect. 3.6.1, Theorem 8), or **(b)** conditioned on that time so that the dwell time in state X is unaffected by the sub-state transition (Sect. 3.6.2, Theorem 9). In both cases, the dwell time distribution for X$_0$ is the minimum of independent Erlang distributions, and sub-states within X$_0$ are as discussed in Sect. 3.5.4. **Panel (a)**: The dwell time in X$_{I_1}$ is Erlang($\varrho _1,\kappa _1$) and independent of time spent in X$_0$. Thus, the sub-state transition alters the overall dwell time in state X. For the more general case, see Sect. 3.6.1 and Theorem 8. **Panel (b)**: The overall X dwell time distribution $T_0\sim$Erlang($r_0,k_0$) is preserved by conditioning the dwell time in the intermediate state on the prior progress through sub-states of X$_0$, as detailed in Sect. 3.6.2. Compare the transitions from X$_0$ to X$_{I_1}$ in these two cases, and recall Fig. 6 and the weak memoryless property of Poisson process first event times from Sect. 3.1.2

Consider the scenario in Fig. 7. Let the X$_0$ dwell time be the minimum of $T_0\sim$Erlang($r_0,2$) and $T_1\sim$Erlang($r_1,2$), with intermediate state dwell time $T_{I_1}\sim$Erlang($\varrho _1,\kappa _1=3$) and an exponential (rate $\mu$) dwell time in Y. Assume the only inputs into X are into X$_0$ at rate ${\mathcal {I}}_X(t)$. By Theorem 8 the corresponding mean field ODEs (see Fig. 8) are Eq. (65), where $x_0(t)=x_{0(1,1)}(t)+x_{0(2,1)}(t)+x_{0(1,2)}(t)+x_{0(2,2)}(t)$ and $x_{I_1}(t)=x_{I_{11}}(t)+x_{I_{12}}(t)+x_{I_{13}}(t)$.

65a $$\begin{aligned} \frac{d}{dt}x_{0(1,1)}(t)&=\; {\mathcal {I}}_X(t) - (r_0+r_1)\,x_{0(1,1)}(t) \end{aligned}$$

65b $$\begin{aligned} \frac{d}{dt}x_{0(2,1)}(t)&=\; r_0 x_{0(1,1)}(t) - (r_0+r_1) x_{0(2,1)}(t) \end{aligned}$$

65c $$\begin{aligned} \frac{d}{dt}x_{0(1,2)}(t)&=\; r_1 x_{0(1,1)}(t) - (r_0+r_1) x_{0(1,2)}(t) \end{aligned}$$

65d $$\begin{aligned} \frac{d}{dt}x_{0(2,2)}(t)&=\; r_0 x_{0(1,2)}(t) + r_1 x_{0(2,1)}(t) - (r_0+r_1) x_{0(2,2)}(t) \end{aligned}$$

65e $$\begin{aligned} \frac{d}{dt}x_{I_{11}}(t)&=\; r_1\,x_{0(1,2)}(t) + r_1\,x_{0(2,2)}(t) - \varrho _1\,x_{I_{11}}(t) \end{aligned}$$

65f $$\begin{aligned} \frac{d}{dt}x_{I_{12}}(t)&=\;\varrho _1\,x_{I_{11}}(t) - \varrho _1\,x_{I_{12}}(t) \end{aligned}$$

65g $$\begin{aligned} \frac{d}{dt}x_{I_{13}}(t)&=\;\varrho _1\,x_{I_{12}}(t) - \varrho _1\,x_{I_{13}}(t) \end{aligned}$$

65h $$\begin{aligned} \frac{d}{dt}y(t)&=\; {\mathcal {I}}_Y(t) + r_0\,x_{0(2,1)}(t) + r_0\,x_{0(2,2)}(t) + \varrho _1\,x_{I_{13}}(t) - \mu \,y(t). \end{aligned}$$

#### Intermediate states that preserve dwell time distributions

In this section we address how to modify the outcome in the previous section to instead construct mean field ODE models that incorporate ‘dwell time neutral’ sub-state transitions, i.e., where the distribution of time spent in X is the same regardless of whether or not particles transition (within X) from some base sub-state X$_0$ to one or more intermediate sub-states X$_{I_j}$. This is done by conditioning the dwell time distributions in X$_{I_i}$ on time spent in X$_0$ in a way that leverages the weak memorylessness property discussed in Sect. 3.1.2.

In applications, this case (in contrast to the previous case) is perhaps the more commonly desired assumption, since modelers often seek to partition states into sub-states where key characteristics, like the overall X dwell time distribution, remain unchanged, but where the different sub-states have functional differences elsewhere in the model. For example, transmission rate reductions from quarantine in SIR type infectious disease models.

One approach to deriving such a model is to condition the dwell time distribution for an intermediate state X$_{I_i}$ on the time already spent in X$_0$ (as in Feng et al. 2016). We take a slightly different approach and exploit the weak memoryless property of Poisson process 1st event time distributions (see Theorem 1 in Sect. 3.1.2, and the notation used in the previous section) to instead condition the dwell time distribution for intermediate states X$_{I_j}$ on how many of the $k_0$ events have already occurred when a particle transitions from X$_0$ to X$_{I_j}$ (rather than conditioning on the exact elapsed time spent in X$_0$). In this case, since each sub-state of X$_0$ has *iid* dwell time distributions that are Poisson process 1st event times with rate $r(t)=\sum _{i=0}^N r_i(t)$, if *i* of the $k_0$ events had occurred prior to the transition out of X$_0$, then the weak memoryless property of Poisson process 1st event time distributions implies that the remaining time spent in X$_{I_j}$ should follow a $(k_0-i)$th event time distribution under an Poisson process with rate $r_0(t)$, thus ensuring that the total time spent in X follows a $k_0$th event time distribution with rate $r_0(t)$. With this realization in hand, one can then apply Theorem 6 and Theorem 4 as in the previous section to obtain the desired mean field ODEs, as detailed in the following Theorem, and as illustrated in Fig. 8.

##### Theorem 9

(Extended LCT with dwell time preserving intermediate states) Consider the mean field equations for a system of particles entering state X (into sub-state X$_0$) at rate ${\mathcal {I}}_X(t)$. As in the previous case, assume the time spent in X$_0$ follows the minimum of $N+1$ independent Poisson process $k_i$th event time distributions with respective rates $r_i(t)$, $i=0,\ldots ,N$ (i.e., $T=\min _i(T_i)$). Particles leaving X$_0$ at time *T* transition to recipient state Y$_\ell$ with probability $p_{0\ell }(T)$ if $T=T_0$, or if $T=T_i$ ($i=1,\ldots ,N$) to the *j*th of $M_X$ intermediate sub-states, X$_{I_j}$, with probability $p_{ij}(T)$. If $T<T_0$, we may define a random variable $K\in \{0,\ldots ,k_0-1\}$ indicating how many events had occurred under the Poisson process associated with $T_0$ at the time of the transition out of X$_0$ (at time *T*). In order to ensure the overall time spent in X follows a Poisson process $k_0$th event time distribution with rate $r_0(t)$, it follows that particles entering state, X$_{I_j}$ will remain there for a duration of time that is conditioned on $K=k$ such that the conditional dwell time for that particle in X$_{I_j}$ will be given by a Poisson process $(k_0-k)$th event time with rate $r_0(t)$. Finally, assume that particles leaving X via intermediate sub-state X$_{I_j}$ at time *t* transition to Y$_\ell$ with probability $q_{j\ell }$, where they remain according to a dwell time determined by survival function $S_\ell (t,\tau )$.

The corresponding mean field equations are

66a $$\begin{aligned}&\frac{d}{dt}x_{0(1,\ldots ,1)}(t) =\; {\mathcal {I}}_X(t) -\sum _{i=0}^{N}r_i(t)\,x_{0(1,\ldots ,1)}(t) \end{aligned}$$

66b $$\begin{aligned}&\frac{d}{dt}x_{0\alpha }(t) =\; \sum _{i=0}^{N} r_i(t)\, x_{0\alpha _{i,-1}}(t)\,\mathbb {1}_{[a_i>1]}(\alpha ) - \sum _{i=0}^{N} r_i(t)\, x_{0\alpha }(t) \end{aligned}$$

66c $$\begin{aligned}&\frac{d}{dt}x_{I_{jk}}(t) =\; r_0(t)\,\big ( x_{I_{j,k-1}}(t)\,\mathbb {1}_{[k>1]} - x_{I_{jk}}(t) \big ) + \sum _{\alpha \in {\mathcal {K}}_{ij}} r_i(t)\,x_\alpha (t)\,p_{ij}(t) \end{aligned}$$

66d $$\begin{aligned}&y_\ell (t) =\; y_\ell (0)S_\ell (t,0) + \int ^t_{0} \bigg ({\mathcal {I}}_{Y_\ell }(\tau ) + \sum _{\alpha \in {\mathcal {K}}_0} r_0(\tau )\,x_\alpha (\tau ) \nonumber \\&\quad + \sum _{j=1}^{M_X} r_0(\tau )\,x_{I_{jk_0}}(\tau )\,q_{j\ell }(\tau ) \bigg ) S_\ell (t,\tau )d\tau \end{aligned}$$

where ${\mathcal {K}}=\{(a_0,a_1,\ldots ,a_N)\;|\;a_j\in \{1,\ldots ,k_j\}\}$, $\alpha \in {\mathcal {K}}{\setminus }(1,\ldots ,1)$, $j=1,\ldots ,M_X$, $k=1,\ldots ,k_0$, $\ell =1,\ldots ,M_Y$, ${\mathcal {K}}_i\subset {\mathcal {K}}$ are the subset of indices where $a_i=k_i$, ${\mathcal {K}}_{ij}\subset {\mathcal {K}}_i$ are the subset of indices where $a_i=k_i$ and $a_0=j$, $x_0(t)=\sum _{\alpha \in {\mathcal {K}}} x_{0\alpha }(t)$, $x_{iI}(t)=\sum _{j=1}^{k_0} x_{iIj}(t)$, and $x(t)=x_0(t)+\sum _{i=1}^{n} x_{iI}(t)$. The $y_\ell (t)$ equations (66d) may be reduced to ODEs, e.g., via Corollary 1.

##### Proof

The proof of Theorem 9 parallels the proof of Theorem 8, but with the following modifications. First, each sub-state of X$_{I_j}$ (for all *j*) has the same dwell time distribution, namely, they are all 1st event time distributions under a Poisson process with rate $r_0(t)$. Second, upon leaving X$_0$ where $T=T_i$ and $K(T)=k < k_0$ (i.e., when only $k<k_0$ events have occurred under the 0th Poisson process; see the definition of *K* in the text above) particles will enter (with probability $p_{ij}(T)$) the *j*th intermediate state X$_{I_j}$ by entering sub-state X$_{I_{jk}}$ which (due to the weak memorylessness property described in Theorem 1) ensures that, upon leaving X$_{I_j}$ particles will have spent a duration of time that follows the Poisson process $k_0$th event time distribution with rate $r_0(t)$. $\square$

##### Example 7

Consider Example 6 in the previous section, but instead assume that the transition to the intermediate state does not impact the overall time spent in state X, as detailed in Theorem 9. The corresponding mean field ODEs for this case are given by Eq. (67) below [compare Eqs. (67e)–(67g) to Eqs. (65e)–(65h)].

67a $$\begin{aligned} \frac{d}{dt}x_{0(1,1)}(t)&=\; {\mathcal {I}}_X(t) - (r_0+r_1)\,x_{0(1,1)}(t) \end{aligned}$$

67b $$\begin{aligned} \frac{d}{dt}x_{0(2,1)}(t)&=\; r_0 x_{0(1,1)}(t) - (r_0+r_1) x_{0(2,1)}(t) \end{aligned}$$

67c $$\begin{aligned} \frac{d}{dt}x_{0(1,2)}(t)&=\; r_1 x_{0(1,1)}(t) - (r_0+r_1) x_{0(1,2)}(t) \end{aligned}$$

67d $$\begin{aligned} \frac{d}{dt}x_{0(2,2)}(t)&=\; r_0 x_{0(1,2)}(t) + r_1 x_{0(2,1)}(t) - (r_0+r_1) x_{0(2,2)}(t) \end{aligned}$$

67e $$\begin{aligned} \frac{d}{dt}x_{I_{11}}(t)&=\; r_1\,x_{0(1,2)}(t) - r_0\,x_{I_{11}}(t) \end{aligned}$$

67f $$\begin{aligned} \frac{d}{dt}x_{I_{12}}(t)&=\; r_1\,x_{0(2,2)}(t) + r_0\,x_{I_{11}}(t) - r_0\,x_{I_{12}}(t) \end{aligned}$$

67g $$\begin{aligned} \frac{d}{dt}y(t)&=\; {\mathcal {I}}_Y(t) + r_0\,x_{0(2,1)}(t) + r_0\,x_{0(2,2)}(t) + r_0\,x_{I_{12}}(t) - \mu \,y(t). \end{aligned}$$

### Generalized Linear Chain Trick (GLCT)

In the preceding sections we have provided various extensions of the Linear Chain Trick (LCT) that describe how the structure of mean field ODE models reflects the assumptions that define corresponding continuous time stochastic state transition models. Each case above can be viewed as a special case of the following more general framework for constructing mean field ODEs, which we refer to as the Generalized Linear Chain Trick (GLCT).

The cases we have addressed thus far share the following stochastic model assumptions, which constitute the major assumptions of the GLCT.

1. A focal state (which we call state X) can be partitioned into a finite number of sub-states (e.g., X$_1,\ldots ,$X$_n$), each with independent (across states and particles) dwell time distributions that are either exponentially distributed with rates $r_i$ or, more generally, are distributed as independent 1st event times under nonhomogeneous Poisson processes with rates $r_i(t)$, $i=1,\ldots ,n$. Recall the equivalence relation in Sect. 3.5.5.
2. Inflow rates into X can be described by non-negative, integrable inflow rates into each of these sub-states (e.g., ${\mathcal {I}}_{X_1}(t),\ldots ,{\mathcal {I}}_{X_n}(t)$), some or all of which may be zero. This includes the case where particles enter X at rate ${\varLambda }(t)$ and are distributed across sub-states X$_i$ according to the probabilities $\varvec{\rho }(t) = [\rho _1(t),\ldots ,\rho _n(t)]^{{\textit{T}}}$ (i.e., we let ${\mathcal {I}}_{X_i}(t)\equiv \rho _i(t)\,{\varLambda }(t)$) where $\sum _i \rho _i =1$.
3. Particles that transition out of a sub-state X$_i$ at time *t* transition either into sub-state X$_j$ with probability $p_{ij}(t)$, or into one of recipient states Y$_\ell$, $\ell =1,\ldots ,m$, with probability $p_{i,n+\ell }$. That is, let $p_{ij}(t)$ denote the probability that a particle leaving state X$_i$ at time *t* enters either X$_j$ if $j\le n$ or Y$_{j-n}$ if $j>n$, where $i=1,\ldots ,n$, $j=1,\ldots ,n,n+1,\ldots ,n+m$.
4. Recipient states Y$_\ell$, $\ell =1,\ldots ,m$, also have dwell time distributions defined by survival functions $S_{Y_\ell }(t,\tau )$ and integrable, non-negative inflow rates ${\mathcal {I}}_{Y_\ell }(t)$ that describe inputs from all other non-X sources.

The GLCT (Theorem 10) describes how to construct mean field ODEs for states X and Y for state transition models satisfying the above assumptions.

#### Theorem 10

(Generalized Linear Chain Trick) Consider a stochastic, continuous time state transition model of particles entering state X and transitioning to states Y$_\ell$, $\ell =1,\ldots ,m$, according to the above assumptions A1-A4. Then the corresponding mean field model is given by

68a $$\begin{aligned} \frac{d}{dt}x_i(t)&=\; {\mathcal {I}}_{X_i}(t) + \sum _{j=1}^n p_{ji}(t)\,r_j(t)\,x_j(t) - r_i(t)\,x_i(t), \quad i=1,\ldots ,n, \end{aligned}$$

68b $$\begin{aligned} y_\ell (t)&=\; y_\ell (0)S_{Y_\ell }(t,0) + \int _{0}^{t} \bigg ( {\mathcal {I}}_{Y_\ell }(\tau ) \nonumber \\&\quad + \sum _{j=1}^{n} r_j(t)\,x_j(\tau )\,p_{j,n+\ell }(t) \bigg ) S_{Y_\ell }(t,\tau )\,d\tau \end{aligned}$$

where $x(t)=\sum _{i=1}^n x_i(t)$, and we assume non-negative initial conditions $x_i(0)=x_{i0}$, $y_\ell (0)=y_{\ell 0}$. Note that the $y_\ell (t)$ equations might be reducible to ODEs, e.g., via Corollary 1 or other results presented above.

Furthermore, Eq. (68a) may be written in vector form where ${\mathbf {P}}_X(t)=(p_{ij}(t))$ ($i,j\in \{1,\ldots ,n\}$) is the $n\times n$ matrix of (potentially time-varying) probabilities describing which transitions out of X$_i$ at time *t* go to X$_j$ (likewise, one can define ${\mathbf {P}}_Y(t)=(p_{ij}(t))$, $i\in \{1,\ldots ,n\}$, $j\in \{n+1,\ldots ,n+m\}$, which is the $n\times m$ matrix of probabilities describing which transitions from X$_i$ at time *t* go to Y$_{j-n}$), $\mathbf {{\mathcal {I}}_X}(t) = [{\mathcal {I}}_{X_1}, \ldots , {\mathcal {I}}_{X_n}]^\text {T}$, ${\mathbf {R}}(t)=[r_1(t),\ldots ,r_n(t)]^\text {T}$, and ${\mathbf {x}}(t)=[x_1(t),\ldots ,x_n(t)]^\text {T}$ which yields

69 $$\begin{aligned} \frac{d}{dt}{\mathbf {x}}(t) =\; \mathbf {{\mathcal {I}}_X}(t) + {\mathbf {P}}_X(t)^\text {T}\,({\mathbf {R}}(t)\circ {\mathbf {x}}(t)) - {\mathbf {R}}(t)\circ {\mathbf {x}}(t). \end{aligned}$$

where $\circ$ indicates the Hadamard (element-wise) product.

#### Proof

The proof of the theorem above follows directly from applying Theorem 4 to each sub-state. $\square$

#### Example 8

**Fig. 9:**
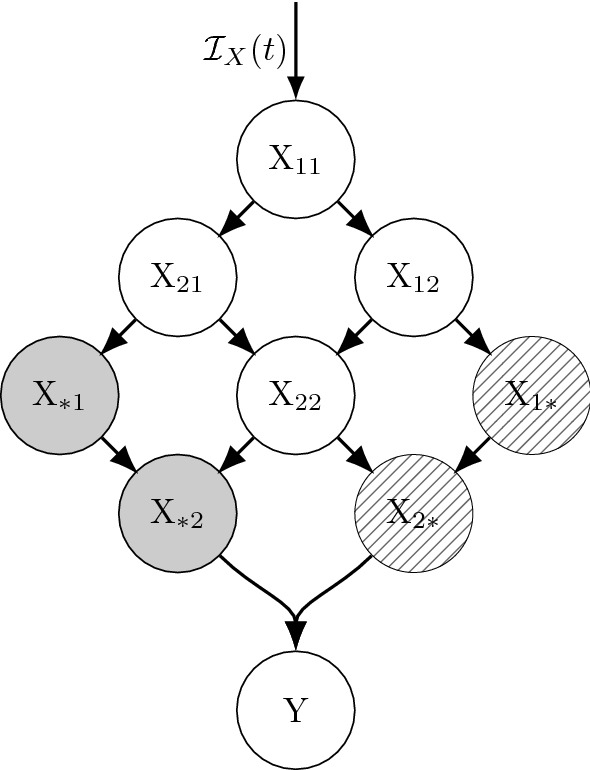
The X sub-state structure for Example 8 where X dwell time distribution follows the maximum of two Erlang random variables with rates $r_1$ and $r_2$, respectively, and shape parameters $k_i=2$. As in Sect. 3.5.4, where the minimum is assumed instead of the maximum, X can be partitioned using indices based on organizing particles by which events they are awaiting under each Poisson process associated with each Erlang distribution. Upon reaching the target event (here, the 2nd event) under any given Poisson process, particles transition to sub-states with an asterisk in the corresponding index position (e.g., see figure). In general, these sub-states all have a dwell times given by the 1st event time under a Poisson process, but with differing rates (see Example 8): here they follow exponential distributions with either rate $r=r_1+r_2$ (white backgrounds), rate $r_2$ (gray backgrounds), or rate $r_1$ (lined backgrounds)

*(Dwell time given by the maximum of independent Erlang random variables)* We here illustrate how the GLCT can provide a conceptually simpler framework for deriving ODEs relative to derivation from mean field integral equations by assuming the X dwell time obeys the maximum of multiple Erlang distributions. While the survival function for this distribution is not straightforward to write down, it is fairly straightforward to construct a Markov Chain that yields such a dwell time distribution (see Fig. 9).

Recall that, in Sect. 3.5.4, we considered a dwell time given by the minimum of *N* Erlang distributions. Here we instead consider the case where the dwell time distribution is given by the maximum of multiple Erlang distributions, $T=\max (T_1,T_2)$ where $T_i\sim$Erlang($r_i,2$). For simplicity, assume the dwell time in a single recipient state Y is exponential with rate $\mu$. We again partition X according to which events (under the two independent homogeneous Poisson processes associated with each of $T_1$ and $T_2$) particles are awaiting, and index those sub-states accordingly (see Fig. 9). These sub-states are X$_{11}$, X$_{21}$, X$_{12}$, X$_{*1}$, X$_{22}$, X$_{1*}$, X$_{*2}$, and X$_{2*}$, where a ‘$*$’ in the *i*th index position indicates that particles in that sub-state have already had the *i*th Poisson process reach the $k_i$th event (in this case, the 2nd event). Each such sub-state has exponentially distributed dwell times, but rates for these dwell time distributions differ (unlike the cases in Sect. 3.5.4 where all sub-states had the same rate): the Poisson process rates for sub-states X$_{11}$, X$_{21}$, X$_{12}$, and X$_{22}$ are $r=r_1+r_2$ (see Fig. 9 and compare to Theorem 6 and Fig. 6), but the rate for the states X$_{1*}$ and X$_{2*}$ (striped circles in Fig. 9) are $r_1$ , and for X$_{*1}$ and X$_{*2}$ (shaded circles in Fig. 9) are $r_2$.

In the context of the GLCT, let ${\mathbf {x}}(t) =$[$x_{11}(t)$, $x_{21}(t)$, $x_{12}(t)$, $x_{*1}(t)$, $x_{22}(t)$, $x_{1*}(t)$, $x_{*2}(t)$, $x_{2*}(t)]^\text {T}$ then by the assumptions above ${\mathbf {R}}(t)=$[*r*, *r*, *r*, $r_2$, *r*, $r_1$, $r_2$, $r_1]^\text {T}$, $\varvec{\rho }(t)=[1,0,\ldots ,0]^\text {T}$ and hence ${\mathcal {I}}_{\mathbf {X}}(t)=[{\mathcal {I}}_X(t),0,\ldots ,0]^\text {T}$. Denote $p_1\equiv r_1/r$ and $p_2\equiv r_2/r$ (*à la* Theorem 7 in Sect. 3.5.5). Then the first eight rows of $9\times 9$ matrix ${\mathbf {P}}$ are given by70Thus, by the GLCT (Theorem 10), the corresponding mean field ODEs are

71a $$\begin{aligned} \frac{d}{dt}x_{11}(t)&=\; {\mathcal {I}}_X(t) - r\,x_{11}(t) \end{aligned}$$

71b $$\begin{aligned} \frac{d}{dt}x_{21}(t)&=\; r_1\,x_{11}(t) - r\,x_{21}(t) \end{aligned}$$

71c $$\begin{aligned} \frac{d}{dt}x_{12}(t)&=\; r_2\,x_{11}(t) - r\,x_{12}(t) \end{aligned}$$

71d $$\begin{aligned} \frac{d}{dt}x_{*1}(t)&=\; r_1\,x_{21}(t) - r_2\,x_{*1}(t) \end{aligned}$$

71e $$\begin{aligned} \frac{d}{dt}x_{22}(t)&=\; r_2\,x_{21}(t) + r_1\,x_{12}(t) - r\,x_{22}(t) \end{aligned}$$

71f $$\begin{aligned} \frac{d}{dt}x_{1*}(t)&=\; r_2\,x_{12}(t) - r_1\,x_{1*}(t) \end{aligned}$$

71g $$\begin{aligned} \frac{d}{dt}x_{*2}(t)&=\; r_2\,x_{*1}(t) + r_1\,x_{22}(t) - r_2\,x_{*2}(t) \end{aligned}$$

71h $$\begin{aligned} \frac{d}{dt}x_{2*}(t)&=\; r_1\,x_{1*}(t) + r_2\,x_{22}(t) - r_1\,x_{2*}(t) \end{aligned}$$

71i $$\begin{aligned} \frac{d}{dt}y(t)&=\; r_1\,x_{2*}(t) + r_2\,x_{*2}(t) - \mu \,y(t). \end{aligned}$$

#### GLCT for phase-type distributions

The GLCT above extends the LCT to a very flexible family of dwell time distributions known as (continuous) *phase-type distributions* (Asmussen et al. 1996; Pérez and Riaño 2006; Osogami and Harchol-Balter 2006; Thummler et al. 2006; Reinecke et al. 2012a; Horváth et al. 2012; Komárková 2012; Horváth et al. 2016; Okamura and Dohi 2015; Horváth and Telek 2017), i.e., the hitting time distributions for Continuous Time Markov Chains (CTMC). These CTMC hitting time distributions include the hypoexponential distributions, hyper-exponential and hyper-Erlang distributions, and generalized Coxian distributions (Reinecke et al. 2012a; Horváth et al. 2016). Importantly, these distributions can be fit to data or can be used to approximate other named distributions (e.g., see Horváth and Telek 2017; Osogami and Harchol-Balter 2006; Altiok 1985; Pérez and Riaño 2006; Komárková 2012; Reinecke et al. 2012b, and related publications). As detailed below, this enables modelers to incorporate a much broader set of dwell time distributions into ODEs than is afforded by the standard LCT.

Consider the assumptions of Theorem 10 above. Assume vectors $\varvec{\rho }(t)=\varvec{\rho }$ and ${\mathbf {R}}(t)={\mathbf {R}}$, and matrices ${\mathbf {P}}_X(t)={\mathbf {P}}_X$, and ${\mathbf {P}}_Y(t)={\mathbf {P}}_Y$ are all constant. As above, assume the probability of entering states in Y is zero, thus our initial distribution vector for this CTMC (with $n+m$ states) is fully determined by the (length *n*) vector $\varvec{\rho }$. Also assume—just to define the CTMC that describes transitions among transient states X up to (but not after) entering states Y—that each state in Y is absorbing (i.e., $p_{ii}=1$, $i>n$). Then the X dwell time distribution follows the hitting time distribution for a CTMC with initial distribution vector $\varvec{\rho }$ and ($n+m$)$\times$($n+m$) transition probability matrix

72 $$\begin{aligned} {\mathbf {P}}=\begin{bmatrix} {\mathbf {P}}_X&\quad {\mathbf {P}}_Y\\ 0&\quad {\mathbf {I}} \\ \end{bmatrix}. \end{aligned}$$

To clearly state the GLCT for phase-type distributions, we must reparameterize the above CTMC. First, there is an equivalent parameterization of this CTMC which corresponds to thinning the underlying Poisson processes so that we only track transitions between distinct states, and ignore when an individual leaves and instantly returns to it’s current state (this thinned process is often called the embedded jump process).

The rate for the thinned process that determines a particle’s dwell time in transient state *i* goes from $r_i$ to

73 $$\begin{aligned} \lambda _i \equiv r_i\,(1-p_{ii}) \end{aligned}$$

If $0 \le p_{ii} < 1$, the transition probabilities out of state *i* then get normalized to

74 $$\begin{aligned} {\widetilde{p}}_{ij} \equiv \frac{p_{ij}}{(1-p_{ii})}, \quad \text {for} \; j\ne i,\text { and } {\widetilde{p}}_{ii}=0. \end{aligned}$$

The rows for absorbing states (Y) remain unchanged, i.e., ${\widetilde{p}}_{ij}=p_{ij}$ for $i>n$. The resulting transition probability matrix $\widetilde{{\mathbf {P}}}$ and rate vector $\varvec{\lambda }$ define the embedded jump process description of the CTMC with transition probability matrix ${\mathbf {P}}$ and rate vector ${\mathbf {R}}$ (initial probability vector $\varvec{\rho }$ is the same for both representations of this CTMC).

Lastly, this CTMC can again be reparameterized by combining the jump process transition probability matrix $\widetilde{{\mathbf {P}}}$ and rate vector $\varvec{\lambda }$ to yield this CTMC’s *transition rate matrix* (also sometimes called the *infinitesimal generator matrix* or simply the *generator matrix*) which is defined as follows. Let matrix ${\mathbf {G}}$ be the same dimension as ${\mathbf {P}}$ (and thus, $\widetilde{{\mathbf {P}}}$) and let the first *n* terms in the diagonal of ${\mathbf {G}}$ be the negative of the jump process rates, $-\varvec{\lambda }$ (i.e., $G_{ii}=-\lambda _i$, $i\le n$). Let the off diagonal entries of the first *n* rows of ${\mathbf {G}}$ be the jump process transition probabilities ${\widetilde{p}}_{ij}$ multiplied by the *i*th rate $\lambda _i$ (i.e., $G_{ij}=\lambda _i\,{\widetilde{p}}_{ij}$, $j\ne i$). Thus, the first row of ${\mathbf {G}}$ is

$$\begin{aligned} {[}\; -\lambda _1 \quad {\widetilde{p}}_{12}\lambda _1 \quad \cdots \quad {\widetilde{p}}_{1n}\lambda _1 \quad \cdots \quad {\widetilde{p}}_{1,n+m}\lambda _1 \; ] \end{aligned}$$

and so on. Since the transition rates out of absorbing states (e.g., the last *m* rows of ${\mathbf {G}}$) are 0, ${\mathbf {G}}$ has the form

75 $$\begin{aligned} {\mathbf {G}}= \begin{bmatrix} {\mathbf {G}}_X&\quad {\mathbf {G}}_Y\\ 0&\quad 0 \\ \end{bmatrix}. \end{aligned}$$

Note that $\widetilde{{\mathbf {P}}}$ and $\varvec{\lambda }$ can be recovered from ${\mathbf {G}}$ using the definitions above.

This third parameterization of the given CTMC, determined solely by initial distribution $\varvec{\rho }$ and transition rate matrix ${\mathbf {G}}$, can now to used to formally describe the phase-type distribution associated with this CTMC, i.e., the distribution of time spent in the transient states X before hitting absorbing states Y. Specifically, the phase-type distribution density function and CDF are

76a $$\begin{aligned} f(t)&=\; \varvec{\rho }^\text {T} \, e^{t\,{\mathbf {G}}_X}(-{\mathbf {G}}_X{\mathbf {1}}) \end{aligned}$$

76b $$\begin{aligned} F(t)&=\; 1 - \varvec{\rho }^\text {T} \, e^{t\,{\mathbf {G}}_X}\,{\mathbf {1}} \end{aligned}$$

where ${\mathbf {1}}$ is a $n\times 1$ vector of ones. Importantly, this distribution depends only on the $n \times n$ matrix ${\mathbf {G}}_X$ and length *n* initial distribution vector $\varvec{\rho }$.

The GLCT for phase-type distributions can now be stated as follows.

##### Corollary 2

(GLCT for phase-type distributions) Assume particles enter state X at rate ${\varLambda }(t)$ and that the dwell time distribution for a state X follows a continuous phase-type distribution given by the $n \times 1$ initial probability vector $\varvec{\rho }$ and $n\times n$ matrix ${\mathbf {G}}_X$. Let ${\mathcal {I}}_{X_i}(t) = \rho _i{\varLambda }(t)$ and $\mathbf {{\mathcal {I}}_X}(t) = [{\mathcal {I}}_{X_1}, \ldots , {\mathcal {I}}_{X_n}]^\text {T}$. Then Eq. (69) in Theorem 10 becomes

77 $$\begin{aligned} \frac{d}{dt}{\mathbf {x}}(t) =\; {\mathcal {I}}_{\mathbf {X}}(t) + {\mathbf {G}}_X^\text {T}\,{\mathbf {x}}(t). \end{aligned}$$

##### Example 9

*(Serial LCT and hypoexponential distributions)* Assume the dwell time in state X is given by the sum of independent (not identically distributed) Erlang distributions or, more generally, Poisson process $k_i$th event time distributions with rates $r_i(t)$, i.e., $T=\sum _i T_i$, $i=1,\ldots ,N$ (note the special case where all $k_i=1$ and $r_i(t)=r_i$ are constant, which yields that *T* follows a hypoexponential distribution). Let $n=\sum _i k_i$ and further assume particles go to $Y_\ell$ with probability $p_{\ell }$ upon leaving X, $\ell =1,\ldots ,m$. Using the GLCT framework above, this corresponds to partitioning X into sub-states X$_j$, where $j=1,\ldots ,n$, and

78 $$\begin{aligned} {\mathbf {R}}(t)=[r_1(t),r_1(t),\ldots ,r_2(t),\ldots ,r_n(t)]^\text {T} \end{aligned}$$

where the first $k_1$ elements of ${\mathbf {R}}(t)$ are $r_1(t)$, the next $k_2$ are $r_2(t)$, etc., and

79 $$\begin{aligned} {\mathbf {P}}_X=\begin{bmatrix} 0&\quad 1&\quad 0&\quad \cdots&\quad 0&\quad 0 \\ 0&\quad 0&\quad 1&\quad \cdots&\quad 0&\quad 0 \\ \vdots&\quad \vdots&\quad \ddots&\quad \ddots&\quad \vdots&\quad \vdots \\ 0&\quad 0&\quad 0&\quad \ddots&\quad 1&\quad 0 \\ 0&\quad 0&\quad 0&\quad \cdots&\quad 0&\quad 1 \\ 0&\quad 0&\quad 0&\quad \cdots&\quad 0&\quad 0 \\ \end{bmatrix}_{n\times n}, \qquad {\mathbf {P}}_Y =\begin{bmatrix} 0&\quad 0&\quad \cdots&\quad 0 \\ \vdots&\quad \vdots&\quad \ddots&\quad \vdots \\ 0&\quad 0&\quad \cdots&\quad 0 \\ p_1&\quad p_2&\quad \cdots&\quad p_m \end{bmatrix}_{n\times \ell }. \end{aligned}$$

By the GLCT (Theorem 10), using $r_{(j)}(t)$ to denote the *j*th element of ${\mathbf {R}}(t)$ in Eq. (78), the corresponding mean field equations are

80a $$\begin{aligned} \frac{d}{dt}x_1(t)&=\; {\mathcal {I}}_X(t) - r_1(t)\,x_1(t) \end{aligned}$$

80b $$\begin{aligned} \frac{d}{dt}x_j(t)&=\; r_{(j-1)}(t)\,x_{j-1}(t) - r_{(j)}(t)\,x_j(t), \text { for } j\ge 1, \end{aligned}$$

80c $$\begin{aligned} y_\ell (t)&=\; y_\ell (0)S_{Y_\ell }(t,0) + \int _{0}^{t} \bigg ( {\mathcal {I}}_{Y_\ell }(\tau ) \nonumber \\&\quad + \sum _{j=1}^{m} r_{(j)}(t)\,x_j(\tau )\,p_{j}(t) \bigg ) S_{Y_\ell }(t,\tau )\,d\tau . \end{aligned}$$

Note, the phase-type distribution form of the hypoexponential distribution could be used with Corollary 2 to derive the $x_i$ equations in Eq. (9).

##### Example 10

*(SIR model with Phase-Type Duration of Infectiousness.)* Consider the simple SIR model given by Eq. (2) with mass action transmission $\lambda (t)=\beta \,I(t)$. Suppose the assumed exponentially distributed dwell times in state *I* were to be replaced by a phase-type distribution with initial distribution vector $\varvec{\alpha }$ and matrix ${\mathbf {A}}$ (note that, in some cases, it is possible to match the first three or more moments using only a $2\times 2$ or $3\times 3$ matrix ${\mathbf {A}}$ and note that Matlab and Python routines for making such estimates are freely available in Horváth and Telek 2017). Then, letting ${\mathbf {x}}$ be the vector of substates of I and $I(t)=x_1(t)+\cdots +x_n(t)$, by the GLCT for phase-type distributions (Corollary 2) the corresponding mean field ODE model, with $R(t)=N_0-S(t)-I(t)$, is

81a $$\begin{aligned} \frac{d}{dt}S(t)&=\; -\beta \,I(t)\,S(t) \end{aligned}$$

81b $$\begin{aligned} \frac{d}{dt}{\mathbf {x}}(t)&=\; \varvec{\alpha }\,\beta \,I(t)\,S(t) + {\mathbf {A}}^\text {T}\,{\mathbf {x}}(t). \end{aligned}$$

## Discussion

ODEs are arguably the most popular framework for modeling continuous time dynamical systems in applications. This is in part due to the relative ease of building, simulating, and analyzing ODE models, which makes them very accessible to a broad range of scientists and mathematicians. The above results generalize the Linear Chain Trick (LCT), and detail how to construct mean field ODE models for a broad range of scenarios found in applications based on explicit individual-level stochastic model assumptions. Our hope is that these contributions improve the speed and efficiency of constructing mean field ODE models, increase the flexibility to make more appropriate dwell time assumptions, and help clarify (for both modelers and those reading the results of their work) how individual-level stochastic assumptions are reflected in the structure of mean field ODE model equations. We have provided multiple novel theorems that describe how to construct such ODEs directly from underlying stochastic model assumptions, without formally deriving them from an explicit stochastic model or from intermediate integral equations. The Erlang distribution recursion relation (Lemma 1) that drives the LCT has been generalized to include the time-varying analogues of Erlang distributions—i.e., *k*th event time distributions under nonhomogeneous Poisson processes (Lemma 2)—and distributions that reflect “competing Poisson process event times” defined as the minimum of a finite number of independent Poisson process event times (Lemma 3). These new lemmas, and our generalization of the memorylessness property of the exponential distribution (which we refer to in Sect. 3.1.2 as the *weak memorylessness* property of nonhomogeneous Poisson process 1st event time distributions) together allow more flexible dwell time assumptions to be incorporated into mean field ODE models. We have also introduced a novel Generalized Linear Chain Trick (GLCT; Theorem 10 in Sect. 3.7) which complements previous extensions of the LCT (e.g., Jacquez and Simon 2002; Diekmann et al. 2017) and allows modelers to construct mean field ODE models for a broad range of dwell time assumptions and associated sub-state configurations (e.g., conditional dwell time distributions for intermediate sub-state transitions), including the phase-type family of distributions and their time-varying analogues as detailed in Sect. 3.7.1. The GLCT also provides a framework for considering other scenarios not specifically addressed by the preceding results, as illustrated by Example 8 in which the dwell time distribution follows the maximum of multiple Erlang distributions.

These results not only provide a framework to incorporate more accurate dwell time distributions into ODE models, but also hopefully encourage more comparative studies, such as Feng et al. (2016), that explore the dynamic and application-specific consequences of incorporating non-Erlang dwell time distributions, and conditional dwell time distributions, into ODE models. More study is needed, for example, comparing integral equation models with non-exponential dwell time distributions and their ODE counterparts based on approximating those distributions with Erlang and phase-type distributions.

The GLCT also permits modelers to incorporate the flexible phase-type distributions (i.e., the hitting-time distributions for Continuous Time Markov Chains) into ODE models. This family of probability distributions includes mixtures of Erlang distributions (a.k.a. hyper-Erlang distributions), the minimum or maximum of multiple Erlang distributions, the hypoexponential distributions, generalized Coxian distributions, and others (Reinecke et al. 2012a; Horváth et al. 2016). While the phase-type distributions are currently mostly unknown to mathematical biologists, they have received some attention in other fields and modelers can take advantage of existing methods that have been developed to fit phase-type distributions to other distributions on ${\mathbb {R}}^+$ and to data (see Sect. 3.7.1 for references). These results provide a flexible framework for approximating dwell time distributions, and incorporating those empirically or analytically derived dwell time distributions into ODE models.

There are some additional considerations, and potential challenges to implementing these results in applications, that are worth addressing. First, the increase in the number of state variables may lead to both computational and analytical challenges, however we have a growing number of tools at our disposal for tackling higher dimensional systems (e.g., van den Driessche and Watmough 2002; Guo et al. 2008; Li and Shuai 2010; Du and Li 2014; Bindel et al. 2014). We intend to explore the costs associated with this increase in dimensionality in future publications. Second, it is tempting to assume that the sub-states resulting from the above theorems correspond to some sort of sub-state structure in the actual system being modeled. This is not necessarily the case, and we should be cautious about interpreting these sub-states as evidence of, e.g., cryptic population structure. Third, some of the above theorems make a simplifying assumption that, either at time $t=0$ or upon entry into X, the initial distribution of particles is only into the first sub-state. This may not be the appropriate assumption to make in some applications, but it is fairly straight forward to modify these these initial condition assumptions within the context of the GLCT. Fourth, it may be appropriate in some applications to avoid mean field models, and instead analyze the stochastic model dynamics directly [e.g., see Allen (2010, 2017) and references therein]. Lastly, the history of successful attempts to garner scientific insights from mean field ODE models seems to suggest that such distributional refinements are unnecessary. However, this is clearly not always the case, as evidenced by the many instances in which modelers have abandoned ODEs and instead opted to use integral equations to model systems with non-Erlang dwell time distributions. We hope these results will facilitate more rigorous comparisons between such models and their simplified and/or ODE counterparts, like Feng et al. (2016) and Piotrowska and Bodnar (2018), to help clarify when the use of these ODE models is warranted.

In closing, these novel extensions of the LCT help clarify how underlying assumptions are reflected in the structure of mean field ODE models, and provide a means for incorporating more flexible dwell time assumptions into mean field ODE models directly from first principles without a need to derive ODEs from stochastic models or intermediate mean field integral equations. The Generalized Linear Chain Trick (GLCT) provides both a conceptual framework for understanding how individual-level stochastic assumptions are reflected in the structure of mean field model equations, and a practical framework for incorporating exact, approximate, or empirically derived dwell time distributions and related assumptions into mean field ODE models.

## Figures and Tables

**Fig. 10:**
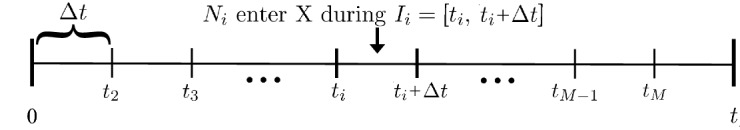
Illustration of discretizing the time interval [0, *t*] into *M* bins (each ${\varDelta } t = t/M$ wide) in order to derive the integral equations (A1b) and (A1c)

## Appendix A Deterministic models as mean field equations

We here give a brief description of the process of deriving deterministic mean field equations from stochastic first principles.

Consider a stochastic model of discrete particles (e.g., individual organisms in a population, molecules in a solution, etc.) that transition among a finite number of *n* states in continuous time. Let the state variables of interest be the amount of particles in each state. This set of counts in each state can be thought of as a *state vector* in the *state space*${\mathbb {N}}^n\subset {\mathbb {R}}^n$, and our model describes the (stochastic) rules governing transitions from one state vector to the next (i.e., from a given state there is some probability distribution across the state space describing how the system will proceed).

Mean field models essentially average that distribution, and thus describe the mean state transitions from any given state of the system. That is, for a given state in ${\mathbb {R}}^n$, we can think of a probability distribution that describes where the system would move from that point in ${\mathbb {R}}^n$, and find the mean transition direction in state space, which then defines a deterministic dynamical system on ${\mathbb {R}}^n$ which we refer to as a mean field model for the given stochastic process. To derive a continuous time mean field model, we do this for an arbitrary discrete step size ${\varDelta } t$ so we can then take the limit as ${\varDelta } t \rightarrow 0$.

### A.1 Deriving mean field integral equations and ODEs

Here we illustrate one approach to derive mean field equations starting from an explicit stochastic model using a simple toy example. To do this, we begin by describing a discrete time approximation (with arbitrarily small time step ${\varDelta } t$) of a stochastic system of particles transitioning among multiple states, then derive the corresponding discrete-time mean field model which then yields the desired mean field model by taking the limit as ${\varDelta } t \rightarrow 0$. Other more systematic approaches exist, e.g., see Kurtz (1970, 1971).

Consider a large number of particles that begin (at time $t=0$) in state W and then each transitions to state X after an exponentially distributed amount of time (with rate *a*). Particles remain in state X according to a Erlang(*r*, *k*) distributed delay before entering state Y. They then go to state Z after an exponentially distributed time delay with rate $\mu$. Let ${{\widetilde{w}}}(t)$, ${\widetilde{x}}(t)$, ${\widetilde{y}}(t)$, and ${\widetilde{z}}(t)$ be the amount in each of the corresponding states at time $t\ge 0$, with $w(0)=N_0$, and $x(0)=y(0)=z(0)=0$. Then, as we will derive below, the corresponding continuous time mean field model is given by Eq. (A1) where the state variables *w*, *x*, *y*, and *z* correspond to the amount in each of the corresponding states, and we assume the initial conditions $w(0)=w_0\gg 1$ and $x(0)=y(0)=z(0)=0$.

A1a $$\begin{aligned} \frac{d}{dt}w(t)\;&=\; -a\,w(t) \end{aligned}$$

A1b $$\begin{aligned} x(t)&=\int _0^t \overbrace{a\,w(s)}^{{\mathcal {I}}(s)}\,\overbrace{S_r^k(t-s)}^{\text {proportion remaining}}\,ds \end{aligned}$$

A1c $$\begin{aligned} y(t)&=\int _0^t\underbrace{\left( \int _0^\tau a\,w(s)\,g_r^k(\tau -s)\,ds\right) }_{\text {Net input rate at time } \tau } S_\mu ^1(t-\tau )\,d\tau \end{aligned}$$

A1d $$\begin{aligned} \frac{d}{dt}z(t)&=\;\mu \,y(t) \end{aligned}$$

First, to derive the linear ODEs (A1a) and (A1d), note that the number of particles that transition from state W to state X in a short time interval $(t,t+{\varDelta } t)$ is binomially distributed: if we think of a transition from W to X as a “success” then the number of “trials” $n={\widetilde{w}}(t)$ and the probability of success *p* is given by the exponential CDF value $p=1-\exp (-a{\varDelta } t)$. For sufficiently small ${\varDelta } t$ this implies $p=a{\varDelta } t+O({\varDelta } t^2)$. Let $w(t+s) = E({\widetilde{w}}(t+s)|{\widetilde{w}}(t))$ for $s\ge 0$. Since the expected value of a binomial random variable is *np* it follows that

A2 $$\begin{aligned} \begin{aligned} w(t+{\varDelta } t) - w(t)&\equiv E({\widetilde{w}}(t+{\varDelta } t) - {\widetilde{w}}(t)|{\widetilde{w}}(t)) \\&= -\, a\,w(t)\,{\varDelta } t + O({\varDelta } t^2). \end{aligned} \end{aligned}$$

Dividing both sides of (A2) by ${\varDelta } t$ and then taking the limit as ${\varDelta } t \rightarrow 0$ yields

A3 $$\begin{aligned} \frac{d}{dt} w(t) = -\,a\,w(t). \end{aligned}$$

Similarly, define *z*(*t*) in terms of ${\widetilde{z}}(t)$ then it follows that

A4 $$\begin{aligned} \frac{d}{dt}z(t)= \;\mu \,y(t). \end{aligned}$$

Next, we derive the integral equations (A1b) and (A1c) by similarly deriving a discrete time mean field model and then taking its limit as bin width ${\varDelta } t \rightarrow 0$ (i.e., as the number of bins $M\rightarrow \infty$).

Partition the interval [0, *t*] into $M\gg 1$ equally wide intervals of width ${\varDelta } t\equiv t/M$ (see Fig. 10). Let $I_i=\big ((i-1){\varDelta } t,i{\varDelta } t\big ]$ denote the *i*th such time interval ($i=1,\ldots ,M$) and let $t_i=(i-1){\varDelta } t$ denote the start time of the *i*th interval. We may now account for the number transitioning into and out of state X during time interval $I_i$, and sum these values to compute *x*(*t*).

The number in state X at time *t* (${\widetilde{x}}(t)$) is the number that entered state X between time 0 and *t*, less the number that transitioned out of X before time *t* (for now, assume $x(0)=0$). A particle that enters state X at time $s\in (0,t)$ will still be in state X according to a Bernoulli random variable with $p=S_{r}^k(t-s)$ (the expected proportion under the given gamma distribution). Therefore, to compute ${\widetilde{x}}(t)$ we can sum over our *M* intervals and add up the number that entered state X during interval $I_i$ and, from each of those *M* cohorts, count how many remain in X at time *t*. Specifically, the number entering X during the *i*th interval [$t,\;t+{\varDelta } t$] is given by $N_i\equiv {\widetilde{w}}(t_i+{\varDelta } t) - {\widetilde{w}}(t_i)$ (see Fig. 10), and thus the number remaining in X at time *t* is the sum of the number remaining at time *t* from each such cohort (i.e., the sum over $i=1$ to *M*) where the number remaining in X at *t* from each cohort follows a compound binomial distribution given by the sum of $N_i$ Bernoulli random variables $B_X$ each with probability $p=S_{r}^k(t-t_i)+O({\varDelta } t)$. This defines our stochastic state transition model, which yields a mean field model as follows.

The expected amount entering X during [$t_i,t_i+{\varDelta } t]$ is $E(N_i)=E({\widetilde{w}}(t_i) - {\widetilde{w}}(t_i+{\varDelta } t))=a\,w(t_i){\varDelta } t$, and the expected proportion of the *i*th cohort remaining at time *t* is $E(B_X)=S_{r}^k(t-t_i) + O({\varDelta } t)$. Thus, the expected number from the *i*th cohort remaining in X at time *t* is $E(N_i)E(B_X)=a\,w(t_i)\,S_{r}^k(t-t_i)\,{\varDelta } t + O({\varDelta } t^2)$. Summing these expected values over all intervals, and letting ${\varDelta } t \rightarrow 0$, yields

A5 $$\begin{aligned} \begin{aligned} x(t) = E({\widetilde{x}}(t))&= \lim _{M\rightarrow \infty } \sum _{i=1}^M a\,w(t_i)\,S_{r}^k(t-t_i)\,{\varDelta } t +O({\varDelta } t^2) \\&=\; \int _0^t a\,w(s)\,S_{r}^k(t-s)\,ds. \end{aligned} \end{aligned}$$

To calculate *y*(*t*), let $K_j$ be the number entering state Y during interval $I_j$ that are still in state Y at time *t*. As above, $K_j$ can be calculated by summing (over $I_1$ to $I_{j-1}$) the number in each cohort that entered state X during $I_i$ then transitioned to state Y during time interval $I_{j}$ and are still in state Y at *t*. Therefore $K_j$ can be written as the sum of $j-1$ compound distributions given by counting how many of the $N_i$ particles that entered state X during $I_i$ then transitioned to state Y during interval $I_j$ and then persisted until time *t* without transitioning to Z. To count these, notice that each such particle entering state X during $I_i$, state Y during $I_j$ and persisting in Y at time *t* follows a Bernoulli random variable $B_{ij}$ with probability

A6 $$\begin{aligned} p_{ij}=\underbrace{(S_r^k(t_j-t_i)-S_r^k(t_{j+1}-t_i)+O({\varDelta } t))}_{\text {P(X}\rightarrow \text {Y in }I_j|W \rightarrow \text {X in } I_i)}\underbrace{(S_\mu ^1(t-t_j)+O({\varDelta } t))}_{\text {P(still in Y at }t)}. \end{aligned}$$

Therefore, the number of particles that entered Y at $I_j$ and remain in state Y at time *t*, $K_j$, can be written as a compound random variable $K_j= \sum _{i=1}^{j-1} \sum _{k=1}^{N_i} B(p_{ij})$. The expected $K_j$ is thus

A7 $$\begin{aligned} \begin{aligned} E(K_j)&= \sum _{i=1}^{j-1} E(N_i) E(B_{ij}) \\&= \sum _{i=1}^{j-1} (a\,w(t_i){\varDelta } t) [(S_r^k(t_j-t_i)-S_r^k(t_{j+1}-t_i))S_\mu ^1(t-t_j)+O({\varDelta } t)] \\&= \sum _{i=1}^{j-1} a\,w(t_i) \frac{(S_r^k(t_j-t_i)-S_r^k(t_j-t_i+{\varDelta } t))}{{\varDelta } t}\,S_\mu ^1(t-t_j)\,{\varDelta } t^2 +O({\varDelta } t^2) \\&= \sum _{i=1}^{j-1} a\,w(t_i) \frac{(G_r^k(t_j-t_i+{\varDelta } t)-G_r^k(t_j-t_i))}{{\varDelta } t}\,S_\mu ^1(t-t_j)\,{\varDelta } t^2 +O({\varDelta } t^2). \end{aligned} \end{aligned}$$

Summing over all intervals and letting ${\varDelta } t \rightarrow 0$ ($M \rightarrow \infty$) gives

A8 $$\begin{aligned} \begin{aligned} y(t)&= \lim _{M\rightarrow \infty } E({\widetilde{y}}(t)) = \lim _{M\rightarrow \infty } E\bigg (\sum _{j=1}^M K_j\bigg ) = \lim _{M\rightarrow \infty } \sum _{j=1}^M E(K_j) \\&=\lim _{M\rightarrow \infty } \sum _{j=1}^M\sum _{i=1}^{j-1} a\,w(t_i) \frac{(G_r^k(t_j-t_i+{\varDelta } t)-G_r^k(t_j-t_i))}{{\varDelta } t}\,{\varDelta } t\\&\quad \cdot S_\mu ^1(t-t_j)\,{\varDelta } t +O({\varDelta } t^2) \\&=\int _0^t\left( \int _0^\tau a\,w(s)\,g_r^k(\tau -s)\,ds\right) S_\mu ^1(t-\tau )\,d\tau . \end{aligned} \end{aligned}$$

Applying Theorem 2, the mean field ODEs equivalent to Eq. (A1), where $x(t)=\sum _{j=1}^kx_j(t)$, are given by

A9a $$\begin{aligned} \frac{d}{dt}w(t)\;&=\; -a\,w(t) \end{aligned}$$

A9b $$\begin{aligned} \frac{d}{dt}{x_1}(t)&=\; a\,w(t) - r\,x_1(t) \end{aligned}$$

A9c $$\begin{aligned} \frac{d}{dt}{x_j}(t)&=\; r\,x_{j-1}(t) - r\,x_j(t), \qquad \text { for } j=2,\ldots ,k \end{aligned}$$

A9d $$\begin{aligned} \frac{d}{dt}{y}(t)&=\; r\,x_{k}(t) - \mu \,y(t) \end{aligned}$$

A9e $$\begin{aligned} \frac{d}{dt}z(t)&=\;\mu \,y(t). \end{aligned}$$

## Appendix B Erlang mixture approximation of Gamma($\alpha ,\beta$)

Here we give a simple example of analytically approximating a gamma distribution with a mixture of two Erlang distributions by moment matching.

Suppose random variable *T* follows a gamma($\alpha ,\beta$) distribution, which has mean $\mu =\beta /\alpha$ and variance $\sigma ^2=\mu /\alpha$, and shape $\beta$ is not an integer. One can approximate this gamma distribution with a mixture of two Erlang distributions that yields the same mean and variance.

These Erlang distributions are T$_{\downarrow }\sim$Erlang($r_{\downarrow },k_{\downarrow }$) and T$_{\uparrow }\sim$Erlang($r_{\uparrow },k_{\uparrow }$) where the shape parameters are obtained by rounding $\beta$ down and up, respectively, to the nearest integer ($k_\downarrow \equiv \lfloor \beta \rfloor$ and $k_\uparrow \equiv \lceil \beta \rceil$) and the rate parameters are given by $r_\downarrow =\alpha \frac{\lfloor \beta \rfloor }{\beta }$ ($r_{\downarrow } = \frac{k_\downarrow }{\mu }$) and $r_\uparrow =\alpha \frac{\lceil \beta \rceil }{\beta }$ ($r_{\uparrow } \equiv \frac{k_\uparrow }{\mu }$). This ensures that T$_{\downarrow }$ and T$_{\uparrow }$ have mean $\mu$.

To calculate their variance, let $p\equiv \lceil \beta \rceil -\beta$ and $q\equiv \beta -\lfloor \beta \rfloor$ (note $p+q=1$). By rounding shape $\beta$ down(up) the resulting Erlang distribution has higher(lower) variance, i.e.,

B1 $$\begin{aligned} \sigma ^2_\downarrow \equiv \frac{\mu }{r_\downarrow }=\sigma ^2 \bigg ( 1+\frac{q}{k_\downarrow } \bigg ) \quad \text { and } \quad \sigma ^2_\uparrow \equiv \frac{\mu }{r_\uparrow }=\sigma ^2 \bigg ( 1-\frac{p}{k_\uparrow } \bigg ). \end{aligned}$$

To calculate the mixing proportion, let the mixture distribution $T_\rho = B_\rho \,T_\downarrow + (1-B_\rho )\,T_\uparrow$, where $B_\rho$ is a Bernoulli random variable with $P(B_p=1)=\rho$ and

B2 $$\begin{aligned} \rho =\frac{p/k_\uparrow }{p/k_\uparrow +q/k_\downarrow }. \end{aligned}$$

This Erlang mixture has the desired mean $E(T_\rho )=\rho \,\mu + (1-\rho )\,\mu =\mu$ and variance $\sigma ^2$, since

B3 $$\begin{aligned} \begin{aligned} \text {Var}(T_\rho )&=\; E\big ((B_\rho \,T_\downarrow + (1-B_\rho )\,T_\uparrow )^2\big ))-\mu ^2\\&=\;E\big ((B_\rho \,T_\downarrow )^2\big )+E\big (((1-B_\rho )\,T_\uparrow )^2\big )-\mu ^2\\&=\;E\big (B_\rho ^2\big )E\big (T_\downarrow ^2\big )+E\big ((1-B_\rho )^2\big )E\big (T_\uparrow ^2\big )-\mu ^2\\&=\;E\big (B_\rho \big )E\big (T_\downarrow ^2\big )+E\big (1-B_\rho \big )E\big (T_\uparrow ^2\big )-\mu ^2\\&=\;\rho \,E\big (T_\downarrow ^2\big )+(1-\rho )\,E\big (T_\uparrow ^2\big )-\mu ^2\\&=\;\rho \,\sigma _\downarrow ^2+(1-\rho )\,\sigma _\uparrow ^2\\&=\;\rho \,\sigma ^2\bigg (1+\frac{q}{k_\downarrow }\bigg )+(1-\rho )\,\sigma ^2\bigg (1-\frac{p}{k_\uparrow }\bigg )\\&=\;\sigma ^2 + \sigma ^2\bigg (\frac{\rho \,q}{k_\downarrow }-\frac{(1-\rho )\,p}{k_\uparrow }\bigg )\\&=\;\sigma ^2 + \sigma ^2\bigg (\frac{p/k_\uparrow \cdot q/k_\downarrow }{p/k_\uparrow +q/k_\downarrow }-\frac{q/k_\downarrow \cdot p/k_\uparrow }{p/k_\uparrow +q/k_\downarrow }\bigg ) = \; \sigma ^2. \end{aligned} \end{aligned}$$

Numerical comparisons suggest this mixture is a very good approximation of the target gamma($\alpha ,\beta$) distribution for shape $\beta$ values larger than roughly 3 to 5, depending (to a lesser extent) on $\alpha$.

Alternatively, to approximate a gamma($\alpha ,\beta$) distribution with a mixture of Erlang distributions as described above, one could also select the mixing probabilities by, for example, using an alternative metric such as a distance in probability space (e.g., see Rachev 1991), e.g., the $L^\infty$-norm on their CDFs, or information-theoretic quantities such as KL or Jensen-Shannon divergence.

## Footnotes

1 Erlang distributions are Gamma distributions with integer-valued shape parameters.

2 They can also be parameterized in terms of their mean and variance (see Appendix B), or with a *shape* and *scale* parameters, where the *scale* parameter is the inverse of the *rate*.

3 A useful interpretation of survival functions, which is used below, is that they give the expected proportion remaining after a give amount time.

4 ...despite the implied exclusivity of the adjective *nonhomogeneous*.

5 That is, the probability of a given individual exiting state X during a brief time period [$t,t+{\varDelta } t$] is approximately $r(t){\varDelta } t$.

6 Here the variance is assumed to have been chosen so that the resulting shape parameter is integer valued. See Appendix B for related details.

7 It is generally true that the survival function for a minimum of multiple independent random variables is the product of their survival functions.
